# When leaders disclose uncertainty: Effects of expressing internal and external uncertainty about a decision

**DOI:** 10.1177/17470218231204350

**Published:** 2023-10-31

**Authors:** Erik Løhre, Karl Halvor Teigen

**Affiliations:** 1 Department of Leadership and Organizational Behaviour, BI Norwegian Business School, Oslo, Norway; 2 Department of Psychology, University of Oslo, Oslo, Norway

**Keywords:** External uncertainty, internal uncertainty, communication, leadership, overconfidence

## Abstract

It is generally assumed that decision-makers appear more competent and trustworthy when exuding confidence in their choices. However, many decisions are by their nature uncertain. Is it possible for a decision-maker to admit uncertainty and still be trusted? We propose that the communicated type of uncertainty may matter. Internal uncertainty, which signals lack of knowledge or a low degree of belief, may be viewed more negatively than external uncertainty, which is associated with randomness and complexity. The results of a series of experiments suggested that people viewed leaders as more competent when they expressed uncertainty about a decision in external (“It is uncertain”) rather than internal terms (“I am uncertain”), overall effect size *d* = 0.45 [0.16, 0.74]. Paradoxically, when asked directly, participants expressed that leaders *should* be open about uncertainty rather than exuding confidence and downplaying uncertainty. A final study suggested that decision makers were more willing to reveal uncertainty about a choice to others when they perceived the uncertainty as more external and less internal and expected more positive and fewer negative consequences from expressing external rather than internal uncertainty.

All decision-makers must routinely confront uncertainty. This creates a dilemma for people in leadership positions: while decision-making in highly uncertain environments is part of their job description (Hoskisson et al., 2017), they are also expected to exude confidence (De Cremer & van Knippenberg, 2004). How then should leaders communicate their decisions: should they always aim to display high confidence, or be open about uncertainty?

In the current article, we investigate two main questions: (1) how leaders who express uncertainty in choice situations are perceived by others, and (2) whether people think leaders *should* be open about uncertainty or rather express confidence. We find that the source of uncertainty may matter: leaders who express uncertainty as an external, objective fact (“it is uncertain”) can be seen as more competent than leaders who express personal, internal uncertainty (“I am uncertain”). However, people state that leaders should be open about uncertainty and rate those who express internal uncertainty as more honest about uncertainty.

## Decision-making, uncertainty and (over)confidence

Uncertainty can occur for many different reasons and is conceptualised in many different ways (Neace et al., 2011; Smithson, 2008). Some conceptualise uncertainty as probability, expressed numerically as a number between 0 and 1, or in words as a verbal probability phrase, for example, “it is likely,” “I am uncertain” (Dhami & Mandel, 2022). Uncertainty about a quantity can be expressed as an interval (e.g., “the sea level will rise between 30 and 60 cm by 2100”), with wide intervals usually associated with higher uncertainty (Løhre et al., 2019). The related concept of confidence refers to beliefs about one’s abilities, knowledge, or skills (Moore & Healy, 2008). In the current studies, we focus on situations with varying degrees of (un)certainty about which among several options is better and the communication of such uncertainty using words. This has relevance for current issues involving a high degree of uncertainty which leaders must decide how to communicate, such as climate change (Kause et al., 2021), COVID-19 (Kelp et al., 2022), and geopolitical risk (Caldara & Iacoviello, 2022).

Implicit leadership theories suggest that prototypical leaders are thought to be industrious, determined, and aggressive (De Cremer & van Knippenberg, 2004; Den Hartog et al., 1999; Hogan & Kaiser, 2005; Ronay et al., 2019). In short, the prototypical leader is thought to be confident, whereas leaders who behave in non-typical ways by revealing uncertainty or lack of confidence may be seen as less competent or less effective and inspire lower motivation and performance among their followers (Bass, 1999; Carless et al., 2000).

Cojuharenco and Karelaia (2020) investigated the perceptions of leaders who ask questions. Participants read about a leader who either presented tentative conclusions (“We can lower costs by reducing the number of suppliers”) or presented the same information in the form of a question (“Can we lower costs by reducing the number of suppliers?”). In both cases, the leader asked for further input on the conclusions/questions. The results showed that if there was already doubt about a leader’s competence, question-asking led to a competence penalty. On the other hand, asking questions also led to a humility premium independent of previous competence beliefs. Thus, leaders who asked questions were seen as less competent, but more humble than leaders who presented tentative conclusions.

Apart from Cojuharenco and Karelaia’s study, few studies investigate perceptions of leaders depending on how or whether they describe the uncertainty associated with their decisions. However, studies in other domains suggest there are advantages of appearing confident: confident advisors are rated as more trustworthy, advisees have a greater tendency to follow advice given with confidence (Sniezek & Van Swol, 2001), and testimony from confident eyewitness are given more weight than testimony from uncertain witnesses (Brewer & Burke, 2002). The higher perceived credibility of confident individuals has been attributed to a “confidence heuristic” (Price & Stone, 2004), where confidence is taken as a cue for competence. Since people generally are more confident about topics they know a lot about, one might assume that speakers who express confidence also are knowledgeable.

The confidence heuristic implies there are benefits of being more confident than justified: even *over*confidence can increase the likelihood of being chosen as an advisor (Radzevick & Moore, 2010), lead to higher social status (C. Anderson et al., 2012) and elevated perceptions of leadership potential (Ronay et al., 2019). Even if the credibility of high-confidence witnesses (Tenney et al., 2007, 2008) and advisors (Sah et al., 2013; Tenney et al., 2019) suffers a greater blow than their low-confidence counterparts when they are revealed to be inaccurate, feedback about accuracy is often unavailable, delayed, or costly, and thus confident advisors often retain their credibility advantage regardless of their accuracy.

These findings suggest that aspiring leaders should try to appear confident. But there may be reasons to be open about uncertainty. First, perceived leadership transparency is associated with greater trust from employees and stakeholders (Norman et al., 2010; Schnackenberg & Tomlinson, 2016), and leaders caught exaggerating confidence or downplaying uncertainty may be seen as less trustworthy. Second, pretending certainty for decisions with large uncertainty is a recipe for organisational overconfidence, with possible negative consequences (Meikle et al., 2016). One important question is whether there are ways to be open about uncertainty that reduce reputational costs.

## Two types of uncertainty

Perceptions of uncertain leaders may depend on how they express uncertainty. A review of research on science communication found that negative effects of communicating scientific uncertainty occurred mostly when uncertainty was operationalised as disagreement or conflict (Gustafson & Rice, 2020). We, however, focus on the well-known distinction between two basic forms of uncertainty in probability theory (Hacking, 1975). *External/aleatory* uncertainty represents external or objective factors, like the indeterminacy of a coin flip, or the causal tendency of a loaded die. *Internal/epistemic* uncertainty is the view that uncertainty is a subjective phenomenon, reflecting degrees of belief or (lack of) knowledge, like the judged probability of an answer to a multiple-choice question being correct. A similar distinction may be reflected in the way people think and talk about uncertainty and probability (Kahneman & Tversky, 1982).

The topic of how expressions suggesting different sources of uncertainty influence thoughts, perceptions, and behaviours has been largely neglected in the literature but has recently been investigated in a small number of studies. Løhre and Teigen (2016) gave participants statements about uncertainty suggesting an internal source (“*I am* X% certain”) or an external source (“*It is* X% certain,” “*There is* an X% probability”). People using external expressions when making a prediction were rated as more knowledgeable and their statements as more informative than people using internal expressions, and a lower external than internal probability was thought necessary to recommend an action. Similarly, Ülkümen et al. (2016) found that different *terms* were differently associated with epistemic and aleatory uncertainty. For instance, “certain” and “confident” were associated with epistemic uncertainty, while “probability” and “chance” were associated with aleatory uncertainty (see also Fox & Irwin, 1998; Juanchich et al., 2017; Teigen & Løhre, 2017). The types of uncertainty reviewed by Gustafson and Rice (2020), except possibly for consensus uncertainty, mainly stemmed from external sources.

The positive effects of (over)confidence and the negative effects of uncertainty may relate mostly to displays of *internal* (un)certainty. For instance, an eyewitness who said “Yes, sir, absolutely. I’m certain of it.” was found more credible than one who said “No, sir, I’m not certain of it.” (Tenney et al., 2007), and accurate advice using the “internal” formulation “Your advisor is X% confident . . .” (Sah et al., 2013) was found to be more credible and persuasive when confidence was high rather than low. In these examples, both the term (“confident,” “certain”) and the pronoun (“I am,” “Your advisor is”) point to internal (un)certainty, and low confidence here may be thought to signal lack of knowledge or competence.

It follows that people may be more accepting of uncertainty if it is communicated to suggest an external source. Gaertig and Simmons (2018) asked participants to forecast future events (sports games, stock prices) after receiving advice about the events. Both advisor confidence (e.g., “I am not sure, but . . .” vs. no mention of confidence) and the uncertainty of the advice itself were varied. Advice uncertainty was for instance manipulated by changing the precision of advice (“the Bucks and Cavaliers will score 207 points [between 197 and 217 points]”) or by making categorical vs. probabilistic predictions (“the Chicago Cubs will win this game” vs. “there is a 57% chance that the Chicago Cubs will win this game”). In line with previous research on the confidence heuristic, advisors who expressed low confidence (i.e., internal uncertainty) were judged negatively. However, there was no corresponding dislike for *uncertain* advice, whether uncertainty was operationalised as an outcome range, a numerical probability, or a verbal probability (i.e., different kinds of external uncertainty). Thus, we propose the following:

*Hypothesis 1.* Leaders will be perceived as less competent when they express uncertainty rather than certainty about a choice, but this competence penalty will be smaller for external than for internal uncertainty.

## A preference for confidence?

While many studies show an interpersonal advantage of appearing confident, less is known about what lay people think decision-makers *should* do when there is uncertainty about a choice: do people think decision-makers should express uncertainty openly, or rather downplay it and display confidence? Existing research points in opposite directions. On the one hand, people value transparency and honesty (Norman et al., 2010; Schnackenberg & Tomlinson, 2016), suggesting they would prefer openness about uncertainty. On the other hand, people value confidence in leaders (Den Hartog et al., 1999; Ronay et al., 2019), suggesting a preference for leaders who communicate certainty rather than doubt. In fact, the “confidence gap” between men and women has been proposed as a reason for the lack of female leaders and gender differences in pay, with (over)confident men coming to higher and higher paid positions than women, who tend to be more modest (Budworth & Mann, 2010; Kay & Shipman, 2014; Sterling et al., 2020).

In a study concerning predictions, Armor et al. (2008) found support for *prescribed optimism*. Participants recommended others to make predictions that were overly optimistic, rather than accurate or pessimistic. In other words, participants acted as if they believed there was some value in being unrealistically optimistic (Shepperd et al., 2015). Our second hypothesis proposes that people will show a similar preference for decision-makers who exude confidence. Again, the type of uncertainty may play a moderating role, such that preference for confidence will be stronger for internal than for external uncertainty.

*Hypothesis 2.* People will prefer decision-makers to exude confidence in choice situations rather than admitting uncertainty, particularly in the case of internal uncertainty.

## The present research

In the first four studies (and one supplemental study), we investigated Hypothesis 1, namely, that leaders who express uncertainty will suffer a “competence penalty” (Cojuharenco & Karelaia, 2020), but that this penalty would be smaller for external than for internal uncertainty. Study 1 described a leader expressing external or internal certainty or uncertainty about a choice between two different options, with participants rating the perceived competence of the leader. Study 2 was a pre-registered replication of the first study, with a larger sample size and a simplified design.

Study 3 investigated a possible moderator for the difference between external and internal uncertainty. Previous research has shown that uncertainty is associated with decision avoidance (C. J. Anderson, 2003), such that under uncertainty, actions like deferring a decision or gathering more information may be seen as more natural than making an immediate choice (Lipshitz & Strauss, 1997). Thus, Study 3 compared competence perceptions for leaders expressing external or internal uncertainty who either made a choice between two available options (replicating Study 1 and Study 2), or instead chose to gather more information. We hypothesised that results in the active choice condition would replicate previous results and in addition higher ratings of competence in the postponed choice condition. Furthermore, Study 3 addressed Hypothesis 2, by asking participants whether they preferred leaders to be honest about uncertainty or to downplay uncertainty and exude confidence.

Study 4 employed a within-subjects design, with participants directly comparing two managers expressing external or internal uncertainty, and rating perceived competence and honesty. An additional study (Study S1, reported in the Supplement), which did not attain the desired level of statistical power, investigated whether internal and external uncertainty might be viewed as more acceptable when a choice is made from many options rather than from just two.

Finally, in Study 5, decision-makers reported on their own willingness to express uncertainty to others in choice situations, depending on the type of uncertainty, shedding light on both hypotheses from the viewpoint of a decision maker rather than from that of an observer.

All studies received approval from NSD—Norwegian centre for research data (reference nr. 204809). We report how we determined the sample size, all data exclusions, all manipulations, and all measures collected in this study (Simmons et al., 2012). Data and materials for all studies, and pre-registrations for Study 2, 3, 4, and S1 can be found on https://osf.io/2pxut/. Data were analysed using jamovi 2.3 (jamovi, 2022).

## Study 1

The goal of Study 1 was to investigate Hypothesis 1, namely that in choice situations, leaders will be perceived more positively when they express external rather than internal uncertainty. Participants in this study rated leaders expressing internal or external (un)certainty about a choice between two options.

### Method

#### Participants

People from the United Kingdom and Ireland were recruited using Prolific and received £1.05 for completing the questionnaire (which also included an unrelated study). Out of 326 participants, 67 who failed an attention check or spent less than 3 min on the survey were excluded from the analysis, leaving 259 participants (172 female, 83 male, 2 other, 2 did not indicate sex), with ages ranging from 18 to 71 years (*M* = 33.8, *SD* = 13.3, one participant did not report age).^1^ No a priori power analysis was performed for this study. We aimed for 60 to 80 participants in each of the four main conditions. A sensitivity analysis using G*Power (Faul et al., 2007) shows that with *n* = 259, the study had 80% power to detect an interaction effect, Cohen’s *f* = 0.17 (η^2^ = 0.028), that is, a medium to small effect.

#### Questionnaires

After giving their informed consent, participants read two scenarios about leaders making a choice between two options. Scenario 1 described a tech company with two ongoing innovation projects, Project A and Project B, which can only afford to invest in one of them. The CEO of the company reads a report about the two projects, and sends an email to the two project groups with a conclusion that varied in different conditions. Specifically, the degree of certainty (“quite uncertain” vs. “quite certain”) and the source of uncertainty (external: “it is” vs. internal: “I am”) differed in a 2 x 2 between-subjects design. To illustrate, the conclusion in the external uncertainty-condition read as follows:

The evaluation report shows that both projects have their pros and cons, and it is quite uncertain which project has the greater promise. However, I have decided that we will invest in Project A.

Thus, the CEO would either state that “it is/I am quite certain” or that “it is/I am quite uncertain” which project has the greater promise. In all cases, the CEO chose Project A. After reading the scenario, participants rated their agreement on 7-point scales (from 1—*Disagree completely*, to 7—*Agree completely*) with the following statements: “The CEO seems competent,” “The CEO seems confident,” “The CEO put a lot of thought into the decision,” “The decision was difficult to make for the CEO,” and “The CEO seems like a good leader.” These five items were intended to measure different aspects of leadership related to the core trait of competence (Fiske et al., 2007). Materials for all studies in this paper are provided in the supplement.

On the next page of the survey participants were reminded of the leader’s statement and were informed about the outcome of the choice, which was either positive or negative (“One year after this decision, after large investments of work and money into Project A, it becomes clear that the project has been a success [failure], and that the investment has [not] paid off.”). With knowledge of the outcome, participants rated their agreement (again on scales from 1 to 7) that it was predictable what the outcome would be, that it was clear that the project could go both ways, that the CEO is responsible for the choice, that the CEO deserves credit/blame for the outcome, and that they would trust the CEO to make good decisions in the future. These questions were included for exploratory purposes and results for the post-outcome ratings are presented in the supplement.

After completing Scenario 1, participants received another scenario with the same structure, describing a Minister of Health in a European country choosing between two different strategies to handle an outbreak of multi-resistant bacteria.

After a final scenario where participants gave their numerical translations of internal and external expressions of (un)certainty (not reported since it is not relevant for the main topic of this article), they responded to an individual difference measure, the intolerance of uncertainty scale (IUS; Carleton et al., 2007). This was included as part of an unrelated study, but we also explored whether responses to leaders expressing (un)certainty correlated with the IUS. We found no evidence for this and report results from the IUS in the supplemental materials.

## Results

We combined ratings of competence, confidence, thoughtfulness, decision difficulty, and leadership for each scenario into two indexes of perceived leadership competence (α = .76 for scenario 1, α = .80 for scenario 2).^2^

**Figure 1.**
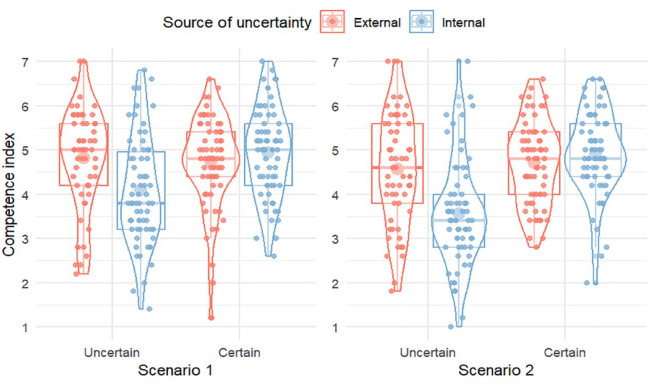
Ratings of leadership competence, by condition and scenario, Study 1. Individual responses are shown as smaller dots. Violins display the distribution of responses. Boxplots display the median, first, and third quartiles. Larger dots show mean values.

A 2 × 2 × 2 mixed analysis of variance (ANOVA) with level of certainty and source of uncertainty as between-subjects factors and scenario as within-subjects factor, showed a main effect of level of certainty, *F*(1, 255) = 21.8, *p* < .001, η_p_^2^ = .079, a main effect of source, *F*(1, 255) = 7.3, *p* = .007, η_p_^2^ = .028, and an interaction between the two factors, *F*(1, 255) = 19.8, *p* < .001, η_p_^2^ = .072. There was additionally a main effect of scenario, *F*(1, 255) = 12.6, *p* < .001, η_p_^2^ = .047, with higher overall ratings in Scenario 1 than in Scenario 2, and an interaction between scenario and level of certainty, *F*(1, 255) = 9.2, *p* = .003, η_p_^2^ = .035, with a larger difference between uncertainty and certainty for scenario 2 (*M*_Diff_ = 0.75) than for scenario 1 (*M*_Diff_ = 0.39), see Figure 1.

As shown in Figure 1, the effect of certainty level was due to higher ratings for “certain” leaders than for “uncertain” leaders, and the effect of source was due to higher ratings for external than for internal expressions. To interpret the interaction, we computed an overall index by taking the average of all 10 items (5 from each scenario), and post hoc tests (Tukey corrected) of main effects on this variable showed a large advantage for external (*M* = 4.69, *SD* = 1.12) over internal uncertainty (*M* = 3.82, *SD* = 1.09), *t*(255) = 4.97, *p* < .001, *d* = 0.89 [0.53, 1.25], while there was no significant difference between external (*M* = 4.72, *SD* = 0.83) and internal certainty (*M* = 4.93, *SD* = 0.86), *t*(255) = −1.26, *p* = .592, *d* = −0.22 [−0.56, 0.12].

### Discussion

In line with previous research, Study 1 showed that certainty is appreciated: leaders were rated as more competent when they expressed certainty rather than uncertainty about a choice. However, leaders suffered a smaller blow to perceived competence when they expressed external rather than internal uncertainty. Although the manipulation of the source of uncertainty was rather subtle (“I am” vs. “it is”), participants were sensitive to the signal that the uncertainty resided either in the world or in the leader himself.

## Study 2

To check the robustness of the findings in Study 1, we ran a higher powered and simplified pre-registered replication. Study 2 used the same scenarios and the same design but did not include information about the outcomes of the decisions and did not include the individual difference measure.

### Method

#### Participants

People from the United Kingdom and Ireland recruited via Prolific received £1.05 for completing the questionnaire (which also included an unrelated study). Thirteen participants who failed an attention check were excluded from the analysis, leaving 632 participants (409 female, 217 male, 6 nonbinary/genderqueer/trans), with ages ranging from 18 to 78 years (*M* = 38.6, *SD* = 12.5, one did not report age).

We pre-registered a sample size of about 600 participants, with a sensitivity analysis using G*Power (Faul et al., 2007) giving 80% power to detect an interaction effect size Cohen’s *f* = 0.11 (η^2^ = 0.012), a small effect.

#### Questionnaire

We used the same materials, design, and procedure as in Study 1, with some minor changes. First, in the scenarios, we removed all references to an “external report,” and instead explained that the leader in each scenario “gathered information” about the options before making a statement. Second, we retained only four questions used from the competence index in Study 1 (perceived competence, confidence, thoughtfulness, and leadership), and excluded the decision difficulty question. Third, we did not include any information about the outcome of the decisions, so participants only rated the leaders on the pre-outcome measures. Fourth, we did not include the intolerance of uncertainty scale.

### Results

**Figure 2.**
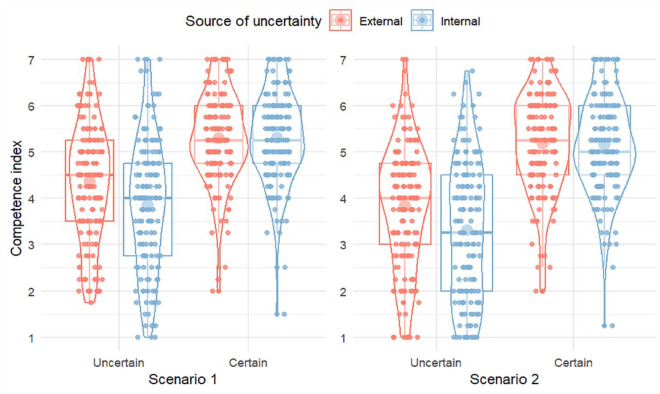
Ratings of leadership competence, by condition and scenario, Study 2. Individual responses are shown as smaller dots. Violins display the distribution of responses. Boxplots display the median, first, and third quartiles. Larger dots show mean values.

The four ratings of the leader had good reliability for both scenario 1 (α = .91) and scenario 2 (α = .94), and averages for each scenario were used as indexes of perceived competence. A 2 × 2 × 2 ANOVA with level of certainty and communicated source as between-subjects factors and scenario as within-subjects factor found a similar pattern of results as in Study 1 (see Figure 2). There was a main effect of level of certainty, *F*(1,628) = 246.42, *p* < .001, η_p_^2^ = .282, a main effect of source, *F*(1,628) = 8.96, *p* = .003, η_p_^2^ = .014, and an interaction between the two factors, *F*(1,628) = 8.61, *p* = .003, η_p_^2^ = .014. In addition, there was a main effect of scenario, *F*(1,628) = 78.93, *p* < .001, η_p_^2^ = .112, and interaction between scenario and level of certainty, *F*(1,628) = 29.64, *p* < .001, η_p_^2^ = .045, with the same pattern as in Study 1 (higher overall ratings for Scenario 1, and larger difference between certainty and uncertainty in Scenario 2 than in Scenario 1). To probe the interaction between source and level of certainty, we computed an overall competence index (average of all items from both scenarios), with post hoc tests (Tukey) showing higher perceived competence for external (*M* = 4.10, *SD* = 1.17) than for internal (*M* = 3.58, *SD* = 1.32) uncertainty, *t*(628) = 4.20, *p* < .001, *d* = 0.47 [0.25, 0.69], while perceived competence was identical for external (*M* = 5.23, *SD* = 0.94) and internal (*M* = 5.22, *SD* = 0.95) certainty, *t*(628) = 0.04, *p* = 1.0, *d* = 0.00 [−0.22, 0.23]. Thus, although the effect size for the difference between internal and external uncertainty in this study was medium rather than large, results were consistent with Study 1.

## Study 3

The results of Study 1 and Study 2 supported Hypothesis 1: leaders were seen as less competent when expressing uncertainty (vs. certainty) about a choice, but this competence penalty was smaller for external than internal expressions of uncertainty. Study 3 introduced a condition where expressing uncertainty may be seen as more natural. Uncertainty is associated with decision avoidance (C. J. Anderson, 2003), and people who wish to delay action will often invoke uncertainty as a reason to postpone, as observed for instance in the context of climate change (Corner et al., 2015). Study 3 compared decision-makers who expressed internal or external uncertainty and either decided on an option (replicating the uncertainty-conditions in Study 1 and 2) or chose to gather more information about the options. We hypothesised that when decision-makers express uncertainty about which option is best and postpone the final choice, they may be seen as more competent than when making a choice despite the uncertainty. We also hypothesised that external uncertainty would lead to higher competence ratings than internal uncertainty when an active choice was made, and perhaps also when the decision was postponed.

Study 3 also investigated what people think leaders should do when there is uncertainty about a choice: should the leader give an honest portrayal of uncertainty, or rather downplay uncertainty and display confidence? Our Hypothesis 2, inspired by findings of prescribed optimism (Armor et al., 2008), was that people would prefer leaders to display confidence, rather than being open about uncertainty, especially for internal uncertainty. We separately pre-registered Part 1 and Part 2 of this study.

### Method

#### Participants

We recruited participants from the United Kingdom and Ireland via Prolific. Those who completed the questionnaire received £0.50. Of 256 people who responded to the survey, 35 were excluded due to failing an attention check, illogical responses to an unrelated study in the same survey, or spending less than 1 min on the survey. The final sample consisted of 221 participants (157 female, 63 male, and 1 other), with ages ranging from 18 to 80 years (*M* = 33.8, *SD* = 12.0).

We pre-registered a sample size of 240 participants, with a sensitivity analysis using G*Power (Faul et al., 2007) giving 80% power to detect an interaction effect of Cohen’s *f* = 0.18 (η^2^ = 0.031).

#### Questionnaires

The scenario described the CEO of a construction company, who was considering two strategies to improve the company’s sustainability: Strategy A, which involved using new experimental building materials, or Strategy B, which involved improving the recycling of materials from building sites. The CEO read a report about the strategies from a group of hired consultants, and then made an announcement, which varied in four different conditions:

It is clear that both strategies have their pros and cons, and based on the report, it is [I am] quite uncertain which project has the greater promise. However, I have decided that we should use Strategy A. [So I have decided that more information should be gathered before I make my choice about which strategy we should use.]

Participants were randomly assigned to one of four conditions in a 2 × 2 between-subjects design (source of uncertainty: internal vs. external; decision type: active choice vs. gather information). The level of uncertainty in this study was kept constant in all conditions. After reading the scenario, participants rated their agreement (from 1—*Disagree completely*, to 7—*Agree completely*) with the same four items as in Study 2.

We next explored how participants thought external or internal uncertainty would change if more information was gathered. Participants in all conditions were informed that the CEO had the consultants gather more information about the two strategies, and that they delivered a new report after 2 months.^3^ Participants then ranked what was most and least likely to happen): that it [the CEO] would become more uncertain, more certain, or that the [CEO’s] uncertainty would not change. Results from this exploratory question are reported in the supplement.

Finally, we asked the participants whether they would prefer a CEO who, after making a choice, communicates that it is [he is] quite uncertain which alternative is better, or a CEO who downplays the [his] uncertainty and communicates that it is [he is] quite certain which alternative is better. Participants were also asked to give a short open-ended explanation (1–3 sentences) of their preference.

## Results

### Competence ratings

As pre-registered, we combined the four ratings into an index of leadership competence, which had high reliability (α = .88).^4^

A 2 × 2 ANOVA on the competence index showed no main effect of source of uncertainty *F* < 1, a main effect of the type of decision, *F*(1,217) = 27.09, *p* < .001, η_p_^2^ = .111, and a non-significant interaction between the two factors, *F*(1,217) = 3.422, *p* = .066, η_p_^2^ = .016. Leaders were rated higher after deciding to gather more information (*M* = 5.22, *SD* = 1.20) than when actively choosing a strategy (*M* = 4.38, *SD* = 1.23), *d* = 0.70 [0.43, 0.97]. This is consistent with an association between uncertainty and “choosing not to choose,” that is, gathering more information is seen as a legitimate approach to try to resolve uncertainty (Lipshitz & Strauss, 1997).

**Figure 3.**
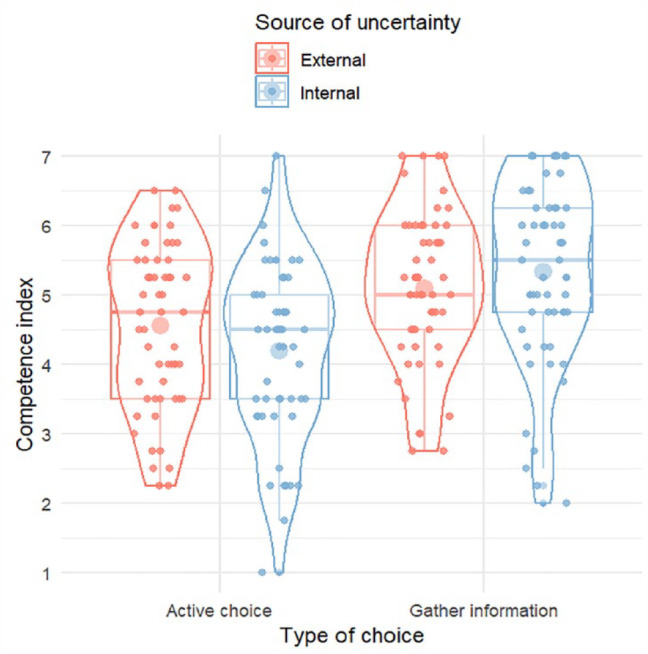
Ratings of leadership competence by condition, Study 3. Individual responses are shown as smaller dots. Violins display the distribution of responses. Boxplots display the median, first, and third quartiles. Larger dots show mean values.

We hypothesised an advantage for external uncertainty in the active choice-condition, while we did not have a strong hypothesis for the information gathering-condition. Probing the non-significant interaction with post hoc tests (Tukey) did not show any significant differences between external and internal uncertainty in any of the two decision type conditions, *p*’s > .40. Figure 3 shows the pattern of results in the different conditions.

### Preference for confidence vs. openness about uncertainty

The participants were asked what they would prefer a CEO to do if he had to make a choice between two alternatives even though he/it was uncertain which alternative is the best: should uncertainty be communicated openly, or should it be downplayed? In both conditions, a majority (67% in the external condition and 58% in the internal condition) declared they would prefer the CEO to communicate uncertainty. Binomial tests showed that the proportion preferring openness about uncertainty was significantly different from 50% in the external condition, *p* < .001, but not in the internal condition, *p* = .101. However, a chi-square test found no significant difference between conditions, χ^2^(1, *N* = 217) = 1.73, *p* = .188. Thus, the results do not support Hypothesis 2 of a preference for confidence, and provide little support for internal vs. external expressions playing a moderating role.

### Discussion

Study 3 showed no statistically significant advantage for external over internal uncertainty in the active choice-condition. Thus, in contrast to the first two studies, Study 3 did not provide support for Hypothesis 1. However, active choice vs. choosing to gather information had a large influence on perceptions of a leader expressing uncertainty, suggesting that expressing uncertainty does not always lead to a competence penalty: both external and internal uncertainty were seen as valid reasons for deferring choice.

Hypothesis 2 was also not supported: when asked whether a leader should appear confident or be open about uncertainty, most participants said they would prefer openness about both internal and external uncertainty. This contrasts with findings that people prescribe overoptimistic predictions (Armor et al., 2008) and with results showing that those who display high confidence are rated as more credible and trustworthy (e.g., Study 1 and 2 in this paper, and studies of the confidence heuristic). Note however that a relatively large minority (33%–42%) preferred a leader to display confidence when making a choice under uncertainty.

## Study 4

The results from the first two studies indicated that the communicated source of uncertainty can have an influence upon how leaders are perceived. An additional study, reported as Study S1 in the supplement since it did not achieve the statistical power we aimed for, showed a non-significant effect of internal vs. external uncertainty on perceptions of competence, and in addition that managers were rated as more honest and open about uncertainty when they used an internal vs. an external expression (*p* = .006, η_p_^2^ = .055).

Study 4 examined the robustness of the effects of external vs. internal uncertainty on perceptions of competence and honesty in a within-subjects design. While different participants rated different expressions in previous studies, in Study 4 all participants could directly compare two leaders who used external and internal uncertainty expressions. In addition, we varied the degree of uncertainty, to see whether potential effects appeared both under high (*very uncertain*) and moderate uncertainty (*somewhat uncertain*). The study was pre-registered on OSF.

### Method

#### Participants

People from the United Kingdom and Ireland were recruited via Prolific and received £0.38 for completing the survey. After excluding eight participants who failed attention checks, had missing responses, or did not finish the survey, there were 187 participants (128 female, 59 male), with ages ranging from 18 to 70 years (*M* = 36.0, *SD* = 12.5).

We preregistered a sample of 180 participants, with a sensitivity analysis using G*Power (Faul et al., 2007) giving 80% power to detect an effect size Cohen’s *d* = 0.21 (for the overall effect of source of uncertainty, independent of the degree of uncertainty).

#### Questionnaires

Participants read a brief description of two managers for different departments in a large tech company who often make decisions and communicate their choices to the employees. They were then given two “slightly different” statements from the two managers, as shown below:

Manager A: “**It is very [somewhat] uncertain** which of the two projects has greater promise. However, I have decided that we will invest in Project X.”

Manager B: “**I am very [somewhat] uncertain** which of the two projects has greater promise. However, I have decided that we will invest in Project Y.”

Thus, Manager A expressed external uncertainty, while Manager B expressed internal uncertainty, and participants were randomly assigned to receive expressions with high (very uncertain) vs. moderate (somewhat uncertain) degree of uncertainty in two between-subjects conditions.

Participants rated which of the two managers seemed more competent, and which of the two managers seemed more honest about a difficult decision (questions presented on separate pages of the survey, in counterbalanced order). The ratings were made on a scale from 1—*Definitely Manager A* to 7—*Definitely Manager B*. Thus, ratings below 4 mean that the manager using an external expression is perceived as more competent/honest, a rating of 4 indicates neutrality, while ratings above 4 indicate that the manager using an internal expression is perceived as more competent/honest. We hypothesised that the manager using an external expression would be rated as more competent but less honest than the manager using an internal expression.

### Results

As shown in Table 1, the results were in line with our hypothesis. One-sample *t*-tests against the midpoint of the scale (4), showed that the manager using an external expression was rated as more competent, *t*(186) = −5.09, *p* < .001, *d* = −0.37 [−0.52, −0.22], while the manager using an internal expression was rated as more honest, *t*(186) = 11.47, *p* < .001, *d* = 0.84 [0.67, 1.00]. Separate one-sample *t* tests for the high and moderate uncertainty conditions gave similar results, and two separate independent samples *t*-tests did not show any significant differences between ratings in the two conditions for competence, *t*(185) = 1.39, *p* = .165, *d* = .20 [−0.09, 0.49] or for honesty, *t*(185) = 1.35, *p* = .177, *d* = .20 [-0.09, 0.49]. There was thus no evidence for a moderating effect of the degree of uncertainty.

**Table 1.** Mean ratings of which manager seems more competent and honest in Study 4, depending on degree of uncertainty (standard deviations in parentheses).

	Somewhat uncertain (*n* = 95)	Very uncertain (*n* = 92)	Overall (*n* = 187)
Competence	3.59 (1.40)	3.28 (1.61)	3.44 (1.51)
Honesty	5.34 (1.30)	5.05 (1.54)	5.20 (1.43)

Ratings below 4 indicate a preference for a manager expressing external uncertainty, while ratings above 4 show a preference for a manager expressing internal uncertainty.

### Discussion

Using a within-subjects design, results in Study 4 were consistent with the overall pattern observed in previous studies. A leader using an external expression of uncertainty was rated as more competent but also less honest and open about uncertainty than a leader using an internal expression of uncertainty. Thus, the effects of using external vs. internal expressions of uncertainty may depend on which of these aspects of leadership (competence or honesty) are more salient at the moment a decision-maker is evaluated.

## Study 5

Studies 1 to 4 involved laypeople evaluating hypothetical decision-makers. In Study 5, we explored the topic in more realistic settings by asking decision-makers about (1) their willingness to express internal or external uncertainty in work situations, and (2) how they thought others would perceive them if they revealed internal or external uncertainty. This examines Hypothesis 2, which states that people believe leaders should exude confidence rather than be open about uncertainty, from the perspective of the decision-maker rather than from an observer.

Study 5 sheds light on the generalisability and practical implications of our findings. If decision-makers are sensitive to the differential effects of internal and external uncertainty, they should be more willing to express external than internal uncertainty and should anticipate fewer negative reputational consequences if they reveal external rather than internal uncertainty.

### Method

#### Participants

We recruited people with management experience via Prolific, by inviting United Kingdom, United States, Ireland, Australia, Canada, or New Zealand nationals who indicated that they (1) had management experience, (2) had authority to instruct subordinates at work, and (3) were in a leadership position/had supervisory duties. Those who completed the survey received £1.13 in compensation. We did not pre-register the study and did not perform a power analysis, but aimed for about 150 participants in each condition.

After excluding 33 participants who failed attention checks, had many missing responses, did not finish the survey, or spent less than 90 s to complete, there were 301 participants (165 female, 136 male), with ages ranging from 19 to 73 years (*M* = 38.4, *SD* = 10.5). Almost all participants reported working full-time (81.4%) or part-time (17.3%). Thirteen percent worked in upper-level management, 40.5% in mid-level management, and 34.6% in lower-level management, with 12% not currently in a management position, but having held one earlier. Participants had on average 8.0 years of management experience (*SD* = 7.6), had authority to give orders to anything from one to more than twenty people (the modal answer, given by 27.6%, being 2–3). Most participants (33.9%) worked in organisations with more than 1000 employees, in a variety of businesses, with IT, retail, health care, government, education, and charity as some examples.

#### Questionnaires

After answering questions about their work experience, participants were randomly assigned to receive either the internal or the external uncertainty version of the questionnaire. First, participants were asked to think about a time “when you as a manager had to make a decision, but you were [it was] uncertain what was the best decision to make” and were told to briefly describe the decision and why they were [it was] uncertain. They were then asked whether they chose to communicate to others that they were uncertain [there was uncertainty], with six options: (1) “I did not mention uncertainty—in fact I explicitly stated that I [it] was quite certain what was the best choice,” (2) “I deliberately chose not to mention that I was uncertain [there was uncertainty],” (3) “I did not find it relevant to mention that I was uncertain [there was uncertainty],” (4) “I mentioned that I was uncertain [there was uncertainty], but downplayed the extent of uncertainty,” (5) “I gave an honest portrayal of how uncertain I [it] was,” (6) “I overstated the degree to which I was uncertain [there was uncertainty].” Later options indicated higher willingness to communicate uncertainty to others.

We expected that participants might not describe situations characterised exclusively by the type of uncertainty they were experimentally assigned to describe. Thus, on the next page of the survey they received four questions measuring how they perceived the uncertainty in the self-described situations, with two questions regarding external uncertainty (e.g., “The uncertainty was an objective fact that would be apparent to other people”) and two questions concerning internal uncertainty (e.g., “The uncertainty was a subjective feeling I had”). Participants indicated their agreement with these statements on scales from 1—*Disagree completely* to 7—*Agree completely*.

Next, participants were asked about their general beliefs about communicating uncertainty to others. They were first asked about their preferences in a situation in which they would “have to make a choice even though you are [it is] uncertain which option is better,” and were given three options: when making my choice I would (1) be open about the fact that I am [it is] uncertain which option is better, (2) not mention the fact that I am [it is] uncertain which option is better, (3) explicitly state that I am [it is] quite certain which option is better.

This was followed on the next page by six statements about the anticipated effects of revealing internal or external uncertainty to others. Participants expressed their agreement (on seven-point scales as above) with statements like “If I reveal that I am uncertain [there is uncertainty] about a choice, I believe that people will see me as incompetent”. The statements were presented in randomised order, and described expectations to be seen as incompetent, honest and open, indecisive, to fail to inspire confidence in others, to prepare others for a variety of outcomes, and to be held less accountable for a potential negative outcome.

Two final questions asked participants to compare internal and external expressions. Specifically, they were asked if they would prefer to say to others when making a choice that “I am uncertain” or that “it is uncertain” which option is the best, and next if they would prefer saying “I am quite certain” or “it is quite certain” which option is best.

### Results

For a specific self-described situation, a majority of participants (52.5%) chose option 5, “I gave an honest portrayal of how uncertain I [it] was,” and only a single participant chose option 6, “I overstated the degree to which I [it] was uncertain,” with the remaining participants spread relatively evenly on options 1, 2, 3 and 4. This is an important finding in itself, and runs counter to Hypothesis 2: there was no preference for exaggerating certainty among decision-makers, instead most claimed to prefer being honest about uncertainty. Based on the distribution of answers, we decided to analyse willingness to communicate uncertainty as a binary variable, with options below 5 indicating low willingness to communicate uncertainty, and options 5 and 6 indicating higher willingness to communicate uncertainty.^5^ In this analysis, participants’ own ratings of type of uncertainty were included as predictors in addition to condition and demographic/work experience variables, since exploratory analyses showed little difference between the type of uncertainty for events in the two conditions, all *t*’s < 1, all *p*’s > .34 (see Table 2).

**Table 2.** Mean agreement ratings (1–7) with statements concerning the type of uncertainty associated with self-described events in Study 5 (standard deviations in parentheses).

	Internal condition (*n* = 152)	External condition (*n* = 147)
External 1: Uncertainty was due to external factors	4.91 (1.69)	4.85 (1.77)
External 2: The uncertainty was an objective fact	4.33 (1.84)	4.51 (1.73)
Internal 1: Uncertainty was related to my knowledge and beliefs	4.22 (1.74)	4.03 (1.76)
Internal 2: The uncertainty was a subjective feeling	3.82 (1.64)	3.68 (1.69)

A logistic regression analysis (Table 3) found no significant effect of condition on willingness to express uncertainty (not surprising given that the perceived type of uncertainty of events did not differ between conditions). However, two of the four ratings of the source of uncertainty predicted willingness to express uncertainty: managers reported higher willingness to express uncertainty the more they experienced uncertainty to be an objective fact but were more hesitant to reveal uncertainty to others the more they experienced uncertainty to be their own subjective feeling. In other words, they were more willing to express uncertainty if they perceived it as more external and less internal. None of the demographic or work experience variables significantly predicted willingness to express uncertainty for this specific, self-described event.

**Table 3.** Logistic regression model for predictors of willingness to communicate uncertainty in a self-described situation, Study 5.

Predictor	*b*	*SE*	Odds ratio	95% CI for odds ratio		*Z*	*p*
				*LL*	*UL*		
Constant	−1.125	0.714	0.325	0.080	1.315	−1.576	.115
Sex^a^	0.313	0.253	1.368	0.833	2.245	1.239	.215
Management experience (in years)	0.029	0.017	1.029	0.996	1.064	1.712	.087
Organisation size^b^	0.019	0.052	1.019	0.920	1.129	0.360	.720
Authority over how many others^c^	−0.041	0.080	0.960	0.821	1.123	−0.511	.609
Condition^d^	0.352	0.244	1.423	0.882	2.295	1.444	.149
External 1: External factors	0.036	0.073	1.037	0.898	1.197	0.496	.620
External 2: Objective fact	0.238	0.072	1.269	1.102	1.460	3.317	<.001
Internal 1: Knowledge/belief	0.038	0.073	1.039	0.900	1.198	0.518	.604
Internal 2: Subjective feeling	−0.174	0.077	0.841	0.723	0.977	−2.256	.024

CI: confidence interval; LL: lower limit; UL: upper limit.

Nagelkerke *R*^2^ = .111, *p* = .002.

a 0 = male, 1 = female.

b 1 = < 20, 7 = > 1000.

c 1 = 1, 6 = > 20.

d 0 = *internal*, 1 = *external*.

When asked about general preferences to express uncertainty, most participants in both conditions said they generally would be open about uncertainty (internal condition – 66.9 %; external condition – 69.1%), and only a small minority said they would prefer to explicitly exaggerate certainty (internal condition – 8.6%; external condition – 9.4%), with the remaining 20%–25% stating they would generally prefer not to mention uncertainty. This result again points in the opposite direction of Hypothesis 2: people say they prefer openness about uncertainty rather than (unwarranted) certainty. We recoded this as a binary variable (openness coded as 1, and the remaining two alternatives as 0), and ran a logistic regression analysis, but none of the predictors had any statistically significant effects on this variable, all *p*’s > .34 (see Table S6 in the Supplementary materials).

Next, we computed two summary scores for participants’ ratings of expected consequences. One consisted of expected negative consequences (to be seen as incompetent and indecisive, and not inspiring confidence in others, α = .89) and one consisted of expected positive consequences (being seen as honest and open, making others better prepared for different outcomes, *r* = .51, *p* < .001), based on the correlation patterns between ratings (Table S7 in the supplement).^6^ As shown in Tables 4 and 5, participants expected less negative and more positive effects of expressing external rather than internal uncertainty. Interestingly, those with more management experience and those who worked in larger organisations were less worried about negative effects of expressing uncertainty and more open for positive effects.

**Table 4.** Linear model for predictors of expected negative consequences of revealing uncertainty, Study 5.

	*b*	*SE*	95% CI		β	*t*	*p*
			*LL*	*UL*			
Constant	4.060	0.292	3.487	4.634		13.925	< .001
Sex^a^	0.096	0.171	−0.242	0.433	0.032	0.558	.577
Management experience (in years)	−0.040	0.011	−0.062	−0.017	−0.199	−3.484	< .001
Organisation size^b^	−0.100	0.036	−0.171	−0.028	−0.159	−2.742	.006
Authority over how many others^c^	0.095	0.055	−0.013	0.203	0.101	1.732	.084
Condition^d^	−0.360	0.169	−0.693	−0.028	−0.120	2.134	.034

CI: confidence interval; LL: lower limit; UL: upper limit.

*R*^2^ = .074, *p* < .001.

a 0 = *male*, 1 = *female*.

b 1 = < 20, 7 = > 1000.

c 1 = 1, 6 = > 20.

d 0 = *internal*, 1 = *external*.

**Table 5.** Linear model for predictors of expected positive consequences of revealing uncertainty, Study 5.

	*b*	*SE*	95% CI		β	*t*	*p*
			*LL*	*UL*			
Constant	5.031	0.213	4.612	5.450		23.623	< .001
Sex^a^	0.079	0.125	−0.167	0.325	0.036	0.631	.528
Management experience (in years)	0.020	0.008	0.004	0.036	0.139	2.404	.017
Organisation size^b^	0.055	0.027	0.003	0.108	0.123	2.090	.037
Authority over how many others^c^	−0.054	0.040	−0.133	0.025	−0.080	−1.354	.177
Condition^d^	0.267	0.123	0.024	0.510	0.123	2.165	.031

CI: confidence interval; LL: lower limit; UL: upper limit.

*R*^2^ = .047, *p* = .013.

a 0 = male, 1 = female.

b 1 = < 20, 7 = > 1000.

c 1 = 1, 6 = > 20.

d 0 = *internal*, 1 = *external*.

Finally, the within-subjects comparison showed that a majority (62%) of the participants preferred to express uncertainty externally, while certainty was preferred to be expressed internally (by 63%). In other words, they would say “it is uncertain” rather than “I am uncertain,” but “I am certain” rather than “it is certain.” Both proportions differed significantly from 50% according to binomial tests, *p*’s < .001.

### Discussion

This study investigating decision-makers’ self-reported willingness to communicate uncertainty resonated with our previous findings. For a specific event chosen by the participants, higher ratings of external and lower ratings of internal uncertainty predicted more willingness to be open about uncertainty. While the type of uncertainty did not influence stated willingness to express uncertainty in general, participants expected more positive and less negative consequences from expressing external uncertainty rather than internal certainty. Furthermore, participants preferred to express uncertainty externally, but certainty internally. Study 5 was not preregistered, and the results should be taken as preliminary evidence suggesting that the difference between internal and external expressions of uncertainty may extend beyond highly controlled hypothetical scenarios.

## General discussion

We started the paper by presenting a dilemma for managers making decisions under uncertainty: how can they admit uncertainty without suffering a blow to their image as competent decision-makers? Our findings suggest it matters how uncertainty is expressed. Results from Study 1, 2, and 4 supported Hypothesis 1, with expressions pointing towards external sources of uncertainty leading to a smaller competence penalty than expressing internal uncertainty.

To get an estimate of the overall effect size across studies (Cumming, 2014), we used the ESCI-module in jamovi (esci in jamovi, 2021). We focused on the effect of the source of uncertainty on perceived competence and included only studies with a between-subjects design with conditions where leaders expressed *un*certainty about a choice between two options and proceeded to make a choice. Thus, the external vs. internal uncertainty conditions from Study 1, Study 2, Study 3 (active choice-condition), and Study S1 (two options-condition) were included. A random-effects internal meta-analysis (*N*_Total_ = 618) showed an overall Cohen’s *d* for the source of uncertainty of 0.45 [0.16, 0.74], with an advantage for external over internal uncertainty, that is, an overall effect of about medium size, but with considerable variation.^7^

Studies 1 and 2 also replicated previous findings of observers using a confidence heuristic: leaders who expressed certainty about a choice were rated as more competent than those who expressed uncertainty but more so for internal than for external expressions. This implies that it is hard to come around the strategic benefits of displaying certainty.

Our second hypothesis proposed that people would “prescribe confidence,” i.e., that they would prefer leaders to exude confidence in choice situations rather than be open about uncertainty. This hypothesis was not supported: in Study 3, the majority stated that leaders should be open about uncertainty, and in Study 5, a majority of decision-makers reported that they would be open about uncertainty both for a specific case and in general. Results were mixed as to whether the source of uncertainty plays a role: only participants’ own ratings of perceived type of uncertainty for a specific event suggested greater willingness to be open about external rather than internal uncertainty. An important limitation is that these results may reflect social desirability concerns. For example, participants in Study 5 might not have wanted to admit that they downplayed uncertainty, but would instead express the socially desirable choice of being honest about uncertainty. Note, however, that 47.5% of the participants still admitted to not fully disclosing uncertainty, and that the final question in Study 5 showed an example of openly self-serving thinking, as participants stated they would prefer to express certainty internally but uncertainty externally (i.e., taking credit for certainty but trying to avoid blame for uncertainty). To circumvent social desirability concerns, future research could measure decision-makers’ communication choices in real time, rather than based on retrospective self-reports. Together, our findings underscore a paradox of communicating uncertainty: when asked directly, people say leaders should be open about uncertainty (Study 3 and 5), but they still give a competence penalty to leaders who express uncertainty rather than certainty about a choice (Studies 1 and 2).

There are situations when a decision-maker may safely express uncertainty. Study 3 showed high competence ratings for a leader who postponed a decision after expressing both internal and external uncertainty. This finding tells us something about common responses to uncertainty. In the domain of climate change, uncertainty has often been used as an excuse to postpone action (Corner et al., 2015; Oreskes & Conway, 2010). Similarly, leaders who mention uncertainty may often do this in the context of reconsidering or pointing towards alternatives that are less uncertain (Lipshitz & Strauss, 1997).

While internal expressions of uncertainty led to a competence penalty as compared to external ones, there was also an “honesty premium” for internal expressions, observed in Study 4 and Study S1. This is potentially important, as perceived leadership transparency is associated with greater trust from employees and stakeholders (Norman et al., 2010; Schnackenberg & Tomlinson, 2016). The opposing effects of internal vs. external uncertainty on competence and honesty perceptions create another dilemma for those who wish to use the current findings strategically for impression management. Whether one should express (internal) certainty about a choice to seem competent, internal uncertainty to seem honest, or external uncertainty as a kind of compromise, probably depends on the specific case and context. Future research could compare these opposing effects for possible downstream consequences, akin to Cojuharenco and Karelaia (2020), who observed that asking questions led to a competence penalty, but that this negative effect could be buffered by a humility premium.

Our studies investigated situations where leaders face a choice between comparable options, what Lipshitz and Strauss (1997) describe as uncertainty due to “undifferentiated alternatives.” There are many other situations where it could be relevant for a decision maker to express uncertainty, and we cannot conclude that expressing external vs. internal uncertainty for instance about predicted consequences, or about the validity or adequacy of information, would be judged similarly. The fact that all studies (except Study 5) in this paper investigated relatively similar scenarios (a leader choosing between different options), is a clear limitation of the present research, and future research should investigate the robustness of these findings in varying contexts. We chose to limit ourselves to this situation because uncertainty at the time of a decision is a highly relevant topic that has received little attention.

In real life, uncertainty is often simultaneously internal and external—we have imperfect knowledge, which can stem from, or come in addition to objective uncertainty. Nevertheless, some situations lend themselves better to an external attribution of uncertainty than others. For instance, few would disagree that the Covid-19 pandemic introduced a large amount of (external) uncertainty into many different domains, often making it very uncertain which choice would be the best (Kerrissey & Edmondson, 2020). However, in times of less volatility, or in situations with well-defined and well-known options, for example, in repeated choice domains, a leader who states that “it is quite uncertain” may be perceived as attempting to avoid responsibility rather than providing an objective assessment (Nordbye et al., 2018). Future research could investigate how decision-makers who inappropriately attribute uncertainty to external factors are perceived, and could also pursue other conceptualisations of uncertainty (Gustafson & Rice, 2020; Smithson, 2008), for instance, whether uncertainty attributed to conflicting opinions or ambiguity has similar effects on leader competence perceptions as internally or externally attributed uncertainty.

The current findings do not provide precise information about why external and internal uncertainty influence perceived competence, but the results are in line with previous related research. Internal uncertainty is associated with a lack of knowledge or degree of belief (Fox & Ülkümen, 2011; Kahneman & Tversky, 1982; Løhre & Teigen, 2016), and accordingly, by expressing internal uncertainty but still making a choice, you imply that you don’t know which option is the best, or don’t have a strong belief in either of the options. Perceivers may then wonder if you shouldn’t have thought more about the decision or gathered more information. On the other hand, the association between external uncertainty and randomness, complexity, or causal tendencies, may lead perceivers to attribute negative reactions in response to expressed uncertainty to the situation rather than the person.

The present studies make several contributions to existing literature on related topics. First, the source of uncertainty may be an overlooked factor in studies of overconfidence and the confidence heuristic. Previous studies seem to have mostly used internally framed language, and our results suggest that external expressions of uncertainty could show a smaller interpersonal advantage for expressing high vs. low certainty. Second, while there have been many studies on how advisors are perceived, there is a lack of studies on the perceptions of decision-makers and leaders. Future studies should further examine decision-makers who need to act on uncertain information, whether and how they express uncertainty, and how they are perceived. Third, while previous studies have mostly focused on degrees of certainty and overconfidence, this study explicitly puts the emphasis on *un*certainty. Although one can express uncertainty by communicating a low degree of certainty, pointing out uncertainty is qualitatively different. A low degree of certainty can be expressed in ways that imply a positive *directionality*, i.e., a focus on an outcome occurring, for instance as “a chance” or as “a 25% probability,” while uncertainty has a negative directionality and is more directly associated with doubt and the possibility that an outcome might not occur (Teigen & Brun, 1995, 1999). Giving more attention to when and how uncertainty is acceptable seems like a valuable approach.

Study 5 indicated that decision-makers who did not want to disclose uncertainty were split between two approaches: staying silent about it or stating that they were certain rather than uncertain. Additional studies should examine the advantages and drawbacks of these options. It seems obvious that lying about certainty is more dishonest than saying nothing. However, we do not know how perceptions of competence will be affected. For instance, the CEO in Studies 1-3 might simply say that “both projects have their pros and cons; however, I have decided that we will invest in Project A,” no mention being made of certainty or uncertainty. Will such a manager be perceived as less confident than one who claims explicitly that he is certain? Competent leaders may not have to express their certainty to become trusted.

## Supplemental Material

sj-docx-1-qjp-10.1177_17470218231204350 – Supplemental material for When leaders disclose uncertainty: Effects of expressing internal and external uncertainty about a decision Supplemental material, sj-docx-1-qjp-10.1177_17470218231204350 for When leaders disclose uncertainty: Effects of expressing internal and external uncertainty about a decision by Erik Løhre and Karl Halvor Teigen in Quarterly Journal of Experimental Psychology

## Notes

1. The number of participants failing the attention check was larger than expected. Results were highly similar when people who failed the attention check were included in the analyses.

2. Ratings of decision difficulty differed somewhat from other ratings and excluding them would have increased reliability (to α = .87 for scenario 1 and α = .88 for scenario 2). However, results are similar whether the decision difficulty variable is included or not.

3. Participants who were first informed that the CEO chose Strategy A were told that the CEO reconsidered and decided it was necessary to gather more information.

4. The ratings of confidence showed a somewhat different pattern than the other ratings. Excluding the confidence ratings gave a larger *p*-value for the interaction (*p* = .121) but did not otherwise substantially change the results, so we report the planned analysis.

5. Treating this variable as continuous and analysing using linear regression gives qualitatively very similar results, indicating that the results are not due to specific analytical choices.

6. Ratings of the expectation to be held less accountable for a potential negative outcome were only weakly related to other ratings and were not included in the analysis. However, if it is included as a positive consequence, results are largely similar.

7. There are several issues with using meta-analysis to claim that an effect is statistically significant overall, see, e.g., Ueno et al. (2016) and Vosgerau et al. (2019). Thus, this internal meta-analysis should be seen as an attempt to give an overall estimate of the effect size in the current set of studies.
