# Supplementary material for: When leaders disclose uncertainty: Effects of expressing internal and external uncertainty about a decision
Source: Q J Exp Psychol (Hove). 2023 Oct 31;77(6):1221–37. doi: 10.1177/17470218231204350 (PMC11134984; doi:10.1177/17470218231204350)
Supplement: sj-docx-1-qjp-10.1177_17470218231204350 – Supplemental material for When leaders disclose uncertainty: Effects of expressing internal and external uncertainty about a decision [file sj-docx-1-qjp-10.1177_17470218231204350.docx]

Supplementary Material for:

**When leaders disclose uncertainty: Effects of expressing internal and external uncertainty about a decision**

Erik Løhre^1^, Karl Halvor Teigen^2^

^1^ Department of Leadership and Organizational Behaviour, BI Norwegian Business School, Oslo, Norway

^2^ Department of Psychology, University of Oslo, Oslo, Norway

*Corresponding author*:

Erik Løhre

BI Norwegian Business School

0442 Oslo

Norway

Telephone: +47 41 42 01 35

E-mail: erik.lohre@gmail.com

Table of contents

[Study 1: additional results 1](#_Toc132635500)

[Study 2: additional results 5](#_Toc132635501)

[Study 3: additional results 5](#_Toc132635502)

[Study 5: additional results 7](#_Toc132635503)

[Supplemental Study S1 8](#_Toc132635504)

[Considerations about pre-registration, error rates, sample size and power analysis 13](#_Toc132635505)

[Materials for all studies 14](#_Toc132635506)

[Study 1 14](#_Toc132635507)

[Study 2 18](#_Toc132635508)

[Study 3 20](#_Toc132635509)

[Study 4 22](#_Toc132635510)

[Study 5 24](#_Toc132635511)

[Supplemental Study S1 32](#_Toc132635512)

[References 35](#_Toc132635513)

# Study 1: additional results

Table S1. *Mean pre-outcome ratings across two scenarios, Study 1. Standard deviations in parentheses*.

|  | Uncertainty | | Certainty | |
| --- | --- | --- | --- | --- |
|  | External  (n = 59) | Internal  (n = 66) | External  (n = 68) | Internal  (n = 66) |
| Competent | 4.51 (1.32) | 3.56 (1.31) | 4.86 (1.02) | 5.02 (1.03) |
| Confident | 4.67 (1.53) | 3.01 (1.46) | 5.72 (0.82) | 5.78 (0.99) |
| Thought | 4.53 (1.35) | 3.60 (1.39) | 4.49 (1.13) | 4.72 (1.09) |
| Decision difficulty | 5.27 (1.21) | 5.49 (1.36) | 3.82 (1.34) | 4.17 (1.33) |
| Leadership | 4.48 (1.33) | 3.45 (1.38) | 4.72 (1.18) | 4.98 (1.07) |
| Competence index | 4.69 (1.12) | 3.82 (1.09) | 4.72 (0.83) | 4.93 (0.86) |

Table S2. *Mean post-outcome ratings across two scenarios for positive outcomes, Study 1. Standard deviations in parentheses*.

|  | Uncertainty | | Certainty | |
| --- | --- | --- | --- | --- |
|  | External  (n = 32) | Internal  (n = 35) | External  (n = 33) | Internal  (n = 35) |
| Predictable | 2.16 (0.98) | 1.96 (0.87) | 3.61 (1.42) | 3.54 (1.09) |
| Both ways | 5.80 (1.11) | 6.06 (1.04) | 4.27 (1.38) | 4.31 (1.22) |
| Responsible | 5.80 (0.92) | 5.63 (1.23) | 5.76 (1.03) | 5.60 (0.98) |
| Credit | 5.14 (1.23) | 4.41 (1.43) | 4.88 (1.31) | 5.29 (1.08) |
| Future trust | 5.03 (1.20) | 4.19 (1.16) | 5.30 (1.04) | 5.39 (0.84) |

Table S3. *Mean post-outcome ratings across two scenarios for negative outcomes, Study 1*. *Standard deviations in parentheses*.

|  | Uncertainty | | Certainty | |
| --- | --- | --- | --- | --- |
|  | External  (n = 27) | Internal  (n = 31) | External  (n = 35) | Internal  (n = 31) |
| Predictable | 2.28 (1.06) | 2.71 (1.06) | 2.57 (1.00) | 2.56 (0.96) |
| Both ways | 5.93 (1.03) | 5.55 (1.10) | 4.39 (1.27) | 3.95 (1.45) |
| Responsible | 5.52 (0.94) | 5.87 (1.22) | 5.37 (1.11) | 5.69 (1.00) |
| Blame | 3.94 (1.71) | 4.61 (1.45) | 4.19 (1.19) | 4.06 (1.12) |
| Future trust | 3.76 (1.57) | 3.05 (1.23) | 3.74 (1.25) | 3.37 (0.96) |

**Pre-outcome ratings.** The main interaction of interest, namely the interaction between level of certainty and source of uncertainty, could also be unpacked by comparing differences between certainty and uncertainty in the internal and external conditions, respectively. Looked at this way, there was no significant difference (with Tukey correction) between certainty and uncertainty in the external condition, *M*_Diff_ = -0.03, *t*(255) = -0.16, *p* = .99, *d* = -0.03 [-0.38, 0.32]. In the internal condition, uncertainty was rated significantly lower than certainty, *M*_Diff_ = -1.11, *t*(255) = -6.52, *p* < . 001, *d* = -1.13 [-1.49, -0.78].

**Post-outcome ratings.** The results for each individual item are displayed in Table S2 and S3 for positive and negative outcomes, respectively. We conducted separate 2 x 2 x 2 ANOVAs for each of the post-outcome variables, with level of certainty (uncertain vs. certain), source of uncertainty (external vs. internal), and outcome of decision (positive vs. negative) as between-subjects factors. For ratings of predictability, there was a main effect of level of certainty, *F*(1,251) = 35.89, *p* < .001, η_p_^2^ = .125, a main effect of outcome, *F*(1,251) = 4.59, *p* = .033, η_p_^2^ = .018, and an interaction between outcome and level of certainty, *F*(1,251) = 29.51, *p* < .001, η_p_^2^ = .105. No other main effects or interactions were significant, *F*’s < 1.7, *p*’s > .19. Positive outcomes were rated as more predictable than negative ones, and ratings of predictability were lower when a leader had expressed uncertainty rather than certainty; the interaction reflects no significant difference between positive and negative outcomes for uncertainty, *M*_Diff_ = -0.44, *t*(251) = -2.28, *p* = .11, but a significant difference when certainty was expressed, *M*_Diff_ = 1.01, *t*(251) = 5.46, *p* < .001.

For ratings of how clear it was that the outcome could go both ways, i.e., “unpredictability”, there was a main effect of certainty level, *F*(1,251) = 112.83, *p* < .001, η_p_^2^ = .310, and marginal interaction between source of uncertainty and outcome, *F*(1,251) = 3.41, *p* = .066, η_p_^2^ = .013. No other effects were statistically significant, all *F*’s < 1.1, all *p*’s > .29. Participants thought it clearer the outcome could go both ways when a leader expressed uncertainty rather than certainty.

Ratings of responsibility were not significantly influenced by either of the experimental factors (or their interaction), with only a marginal interaction between source of uncertainty and outcome, *F*(1,251) = 3.56, *p* = .060, η_p_^2^ = .014, all other *F*’s < 1, all *p*’s > .46. This perhaps reflects a belief that a leader bears the responsibility for the decision he made no matter how certain or uncertain he/it was, and no matter the outcome.

Ratings of credit and blame were influenced by the outcome, *F*(1,251) = 19.71, *p* < .001, η_p_^2^ = .073, with more credit given for positive outcomes than blame for negative outcomes. There was also a three-way interaction, *F*(1,251) = 8.60, *p* = .001, η_p_^2^ = .033, which we explored by conducting separate 2 x 2 ANOVAs for positive and negative outcomes. When the outcome was positive, there was a significant interaction between level of certainty and source of uncertainty, *F*(1,131) = 6.749, *p* = .010, η_p_^2^ = .049. Post hoc tests (Tukey) showed that this interaction was due to people giving the leader slightly, but not significantly, more credit under external uncertainty than external certainty, *t*(131) = 0.83, *p* = .838; while the leader got significantly less credit when he was internally uncertain rather than certain, *t*(131) = -1.88, *p* = .024, *d* = -0.69 [-1.17, -0.21]. When the outcome was negative, there were no statistically significant effects on ratings of blame, all *F*’s < 2.6, all *p*’s > .11. Note that it is not in line with the “calibration hypothesis” (Tenney et al., 2008) that an internally (or externally) certain leader is not “punished” more when the outcome does not go according to plan.

Finally, we analyzed ratings of whether one would trust the CEO to make decisions in the future. A 2 x 2 x 2 ANOVA on this variable showed main effects of source, *F*(1,251) = 10.12, *p* = .002, η_p_^2^ = .039, of level of certainty, *F*(1,251) = 9.39, *p* = .002, η_p_^2^ = .036, and of outcome, *F*(1,251) = 106.39, *p* < .001, η_p_^2^ = .298, and additionally interactions between source and level of certainty, *F*(1,251) = 4.77, *p* = .030, η_p_^2^ = .019, and between outcome and certainty level, *F*(1,251) = 4.04, *p* = .046, η_p_^2^ = .016. Participants reported higher trust for external expression, for high levels of certainty, and when the outcome was positive. The interaction between source and level of certainty was due to higher ratings of trust for external vs. internal uncertainty, *M*_Diff_ = 0.78, *t*(251) = 3.72, *p* = .001, *d* = 0.67 [0.33, 1.03], but little difference between external and internal certainty, *M*_Diff_ = 0.14, *t*(251) = 0.72, *p* = .89, *d* = 0.12 [-0.22, 0.74]. The interaction between outcome and certainty level was due to less trust to uncertainty than certainty when the outcome was positive, *M*_Diff_ = -0.74, *t*(251) = -3.67, *p* = .002, *d* = -0.63 [-0.98, -0.29], but no difference in trust between uncertainty and certainty when the outcome was negative, *M*_Diff_ = -0.15, *t*(251) = -0.83, *p* = .885, *d* = -0.13 [-0.49, 0.22].

**Intolerance of uncertainty.** The 12 items of the short Intolerance of Uncertainty Scale (IUS) had high internal consistency (α = .90), and a sum score was computed for each participant. The scores were approximately normally distributed, with a mean of 38.3 (*SD* = 9.2), and a 2 x 2 x 2 ANOVA with the experimental conditions as between-subjects factors showed no significant differences between IUS-scores in the different conditions (all *F*’s < 2, all *p*’s > .16).

Including the IUS score as a covariate in the ANOVAs reported above did not substantially change the results for neither pre- nor post-outcome ratings. Furthermore, correlational analyses did not find any clear overall patterns. IUS showed a statistically significant correlation with ratings of how difficult it was for the leader to make a decision, *r* = .177, *p* = .004, and with ratings of how good the leader was, *r* = .145, *p* = .020, all other *r*’s < .12, all *p*’s > .06; for the post-outcome ratings, there were no statistically significant correlations overall, all *r*’s < .11 all *p*’s > .10. Inspecting correlations separately for each condition similarly did not reveal any clear patterns. Thus, overall the results did not appear to be influenced by individual differences in intolerance of uncertainty.

# Study 2: additional results

Table S4. *Mean ratings across two scenarios, Study 2. Standard deviations in parentheses*.

|  | Uncertainty | | Certainty | |
| --- | --- | --- | --- | --- |
|  | External  (n = 158) | Internal  (n = 159) | External  (n = 159) | Internal  (n = 156) |
| Competent | 4.08 (1.20) | 3.57 (1.40) | 5.06 (1.10) | 5.10 (1.07) |
| Confident | 4.18 (1.35) | 3.31 (1.53) | 5.75 (1.06) | 5.76 (1.02) |
| Thought | 4.15 (1.36) | 3.84 (1.49) | 5.10 (1.12) | 5.08 (1.10) |
| Leadership | 4.01 (1.20) | 3.60 (1.39) | 5.02 (1.01) | 4.96 (1.06) |
| Competence index | 4.10 (1.17) | 3.58 (1.32) | 5.23 (0.94) | 5.22 (0.95) |

We also analyzed the interaction between level of certainty and source of uncertainty by comparing differences between certainty and uncertainty in the internal and external conditions, respectively. Looked at this way, post hoc tests (Tukey) showed that leaders were rated significantly less competent when expressing uncertainty rather than certainty, both in the external condition, *M*_Diff_ = -1.13, *t*(628) = -9.04, *p* < .001, *d* = -1.02 [-1.24, -0.79], and in the internal condition, *M*_Diff_ = -1.64, *t*(628) = -12.15, *p* < . 001, *d* = -1.48 [-1.72, -1.25].

# Study 3: additional results

Table S5. *Mean ratings of a CEO depending on expressed source of uncertainty and type of choice, Study 3. Standard deviations in parentheses.*

|  | Active choice | | Gather information | |
| --- | --- | --- | --- | --- |
|  | External  (n = 56) | Internal  (n = 53) | External  (n = 55) | Internal  (n = 57) |
| Competent | 4.59 (1.23) | 4.09 (1.26) | 5.35 (1.09) | 5.47 (1.45) |
| Confident | 4.80 (1.61) | 4.23 (1.71) | 4.53 (1.51) | 4.81 (1.66) |
| Thought | 4.38 (1.59) | 4.15 (1.56) | 5.33 (1.38) | 5.72 (1.28) |
| Leadership | 4.45 (1.33) | 4.28 (1.42) | 5.20 (1.15) | 5.35 (1.38) |
| Competence index | 4.55 (1.18) | 4.19 (1.26) | 5.10 (1.10) | 5.34 (1.29) |

**Does added information change uncertainty?** After rating the competence of the decision maker in Study 3, participants were asked what they thought would happen to external or internal uncertainty if more information was gathered. Did they think that uncertainty would increase, decrease, or stay the same? As Figure S1 makes evident, the pattern of rankings was similar in both conditions: participants thought it most likely that more information would increase certainty, next most likely that the uncertainty would stay the same, and least likely that more information would lead to more uncertainty. However, separate Mann-Whitney tests showed that certainty was rated as slightly more likely to increase in the internal than in the external condition, *U* = 4181, *p* = .027, *r* = .13, while uncertainty was rated as less likely to stay the same in the internal as compared to the external condition, *U* = 3842, *p* = .003, *r* = .20. There was no difference in the rated likelihood of increasing uncertainty between conditions, *U* = 4483, *p* = .341.^[[1]](#footnote-1)^ In other words, the results indicate that gathering information is expected to especially increase internal certainty, while external uncertainty is seen as more likely to stay the same.


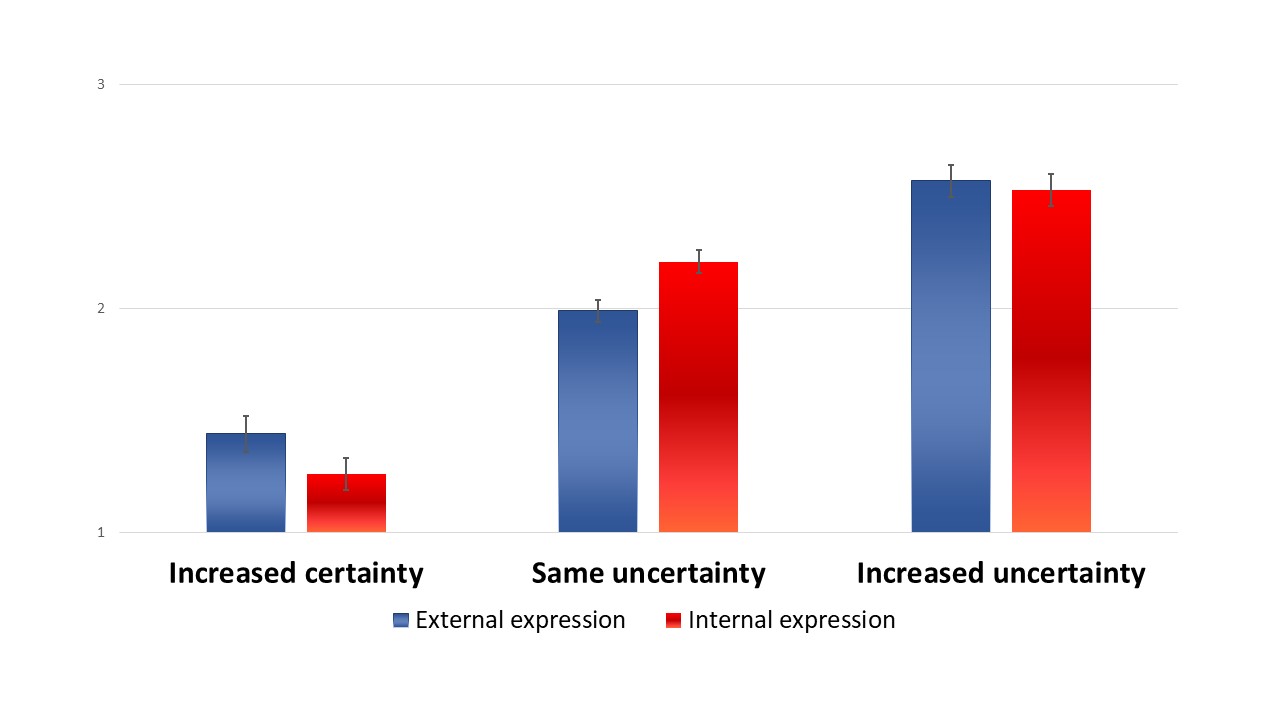


*Figure S1*. Mean rank assigned to the likelihood of information leading to more uncertainty, more certainty, or no change in the level of uncertainty, Study 3. Error bars: ± 1 SEM.

# Study 5: additional results

Table S6. *Logistic regression model for predictors of willingness to communicate uncertainty in general, Study 5. 95% confidence intervals for b reported in brackets.*

|  | *b* | *SE for b* | Odds ratio | *Z* | *p* |
| --- | --- | --- | --- | --- | --- |
| Constant | 0.642 | 0.426 | 1.90  [0.82, 4.38] | 1.506 | .132 |
| Sex  (0 = male, 1 = female) | 0.031 | 0.252 | 1.03  [0.63, 1.69] | 0.123 | .902 |
| Management experience (in years) | 0.007 | 0.017 | 1.01  [0.97, 1.04] | 0.400 | .689 |
| Organization size  (1 = < 20, 7 = > 1000) | 0.050 | 0.054 | 1.05  [0.95, 1.17] | 0.938 | .348 |
| Authority over how many others  (1 = 1, 6 = >20) | -0.066 | 0.081 | 0.936  [0.80, 1.10] | -0.816 | .415 |
| Condition  (0 = internal, 1 = external) | 0.104 | 0.249 | 1.109  [0.68, 1.81] | 0.416 | .677 |

*Note: Nagelkerke R*^2^ = 0.007, *p* = .911

Table S7. *Descriptive statistics and Pearson correlations between ratings of expected consequences of revealing uncertainty to others, Study 4.*

| Variable | *n* | *M* | *SD* | 1 | 2 | 3 | 4 | 5 | 6 |
| --- | --- | --- | --- | --- | --- | --- | --- | --- | --- |
| 1. Incompetent | 301 | 3.05 | 1.64 | — |  |  |  |  |  |
| 2. Indecisive | 301 | 3.74 | 1.72 | .74^***^ | — |  |  |  |  |
| 3. Failing to inspire confidence | 301 | 3.74 | 1.65 | .73^***^ | .71^***^ | — |  |  |  |
| 4. Honest and open | 301 | 5.55 | 1.24 | -.49^***^ | −.39^***^ | -.45^***^ | — |  |  |
| 5. Preparing others for different outcomes | 301 | 5.30 | 1.26 | −.21^***^ | -.18^**^ | -.20^***^ | .51^***^ | — |  |
| 6. Being held less accountable for a negative outcome | 300 | 3.35 | 1.59 | .09 | .16^**^ | .12^*^ | .04 | .14^*^ | — |

* *p* < .05, ** *p* < .01, *** *p* < .001

# Supplemental Study S1

Supplemental Study S1 investigated another potential moderator for the effect of the source of uncertainty on perceptions of competence, namely the number of options that a leader is choosing between. Several studies show that decision difficulty may increase when the number of options increases (Chernev et al., 2015), and it is natural that uncertainty may also increase as the choice set gets larger. Hence, people may find it more natural for a leader to express uncertainty about a choice when options are numerous than when there are two alternatives. Thus, Study S1 compared perceptions of leaders that express external vs. internal uncertainty in a choice situation with two vs. multiple options. In addition to asking about perceived competence, we also included questions about how honest and open about uncertainty the leader seemed to be. The study was pre-registered on [OSF](https://osf.io/hmbzv/?view_only=ae2ed8dfd380432b85b19622b8a49343).

**Method**

**Participants**. Students from two Norwegian universities were asked to participate as volunteers. After excluding 35 participants who failed the attention checks, or did not finish the survey, there were 139 participants^[[2]](#footnote-2)^ (102 female, 37 male), with ages ranging from 20 to 44 years (*M* = 23.4, *SD* = 4.0, one did not indicate age).

We aimed for about 45 participants per condition (i.e., a total of 180 participants), with a sensitivity analysis using G*Power (Faul et al., 2007) showing that this would give a power of 0.8 to detect an effect of Cohen’s *f* = 0.21 (η^2^ = 0.042). The actual sample size (n = 139) gives a power of 0.8 to detect an effect of Cohen’s *f* = 0.24 (η^2^ = 0.054).

**Questionnaires.** There were two scenarios describing a leader making a choice between options. In both scenarios, the leader orders a report describing all available options, before announcing his choice. To illustrate, the second scenario described a leader of a marketing department choosing which new product to make a TV-commercial about:

*“Both of the two products [All of the eight products] have potential, but based on the report, it is [I am] quite uncertain which product would benefit most from a TV-commercial. However, I have decided that we should make a TV-commercial for Product A.”*

Participants were randomly assigned to different conditions in a 2 x 2 between-subjects design, with the source of uncertainty either expressed as internal (“I am quite uncertain”) or external (“it is quite uncertain”), and the number of options were either few (two) or many (six in scenario one, eight in scenario two). After reading each scenario, the participants rated their agreement with the same four items that were used for the competence index in the previous studies.

After the second scenario, we included three questions to explore how expressing external or internal uncertainty would relate to perceptions of honesty and openness about uncertainty, and about the reason behind the choice. Using seven-point scales from 1 (Disagree completely) to 7 (Agree completely), participants indicated whether they thought the leader seemed honest about difficult decisions, whether the leader seemed unafraid to admit uncertainty (we label this “openness”), and whether the leader provided a good reason for his choice. We had no clear hypotheses for these questions, other than expecting that the questions about honesty and openness would be related.

Table S8. *Mean ratings of leaders across two scenarios depending on expressed source of uncertainty and number of options, Supplemental Study S1. Standard deviations in parentheses.*

|  | Two options | | Multiple options | |
| --- | --- | --- | --- | --- |
|  | External  (n = 34) | Internal  (n = 33) | External  (n = 39) | Internal  (n = 33) |
| Competent | 3.41 (1.40) | 3.24 (1.36) | 3.87 (1.49) | 3.15 (1.45) |
| Confident | 3.01 (1.57) | 2.79 (1.33) | 4.31 (1.75) | 2.76 (1.52) |
| Thought | 3.07 (1.62) | 3.14 (1.35) | 3.65 (1.61) | 3.24 (1.55) |
| Leadership | 3.40 (1.43) | 3.41 (1.42) | 3.78 (1.41) | 3.36 (1.43) |
| Competence index | 3.22 (1.33) | 3.14 (1.12) | 3.90 (1.40) | 3.13 (1.23) |

**Results**

Table S7 shows an overview of the results. We followed our pre-registered approach and combined the eight ratings (four per scenario) into an index of perceived leadership competence (α = .91)^[[3]](#footnote-3)^. A 2 x 2 ANOVA found that the main effect of source of uncertainty on the overall ratings of leadership competence was not statistically significant, *F*(1,135) = 3.86, *p* = .051, η_p_^2^ = .028, even if descriptively ratings were somewhat higher for external (*M* = 3.59, *SD* = 1.40) than for internal (*M* = 3.14, *SD* = 1.16) uncertainty, *d* = 0.33 [-0.00, 0.67] There was no statistically significant effect of number of options, *F*(1,135) = 2.33, *p* = .129, η_p_^2^ = .017, nor any interaction between the two factors, *F*(1,135) = 2.55, *p* = .113, η_p_^2^ = .019. Figure S2 shows the results for the competence index.


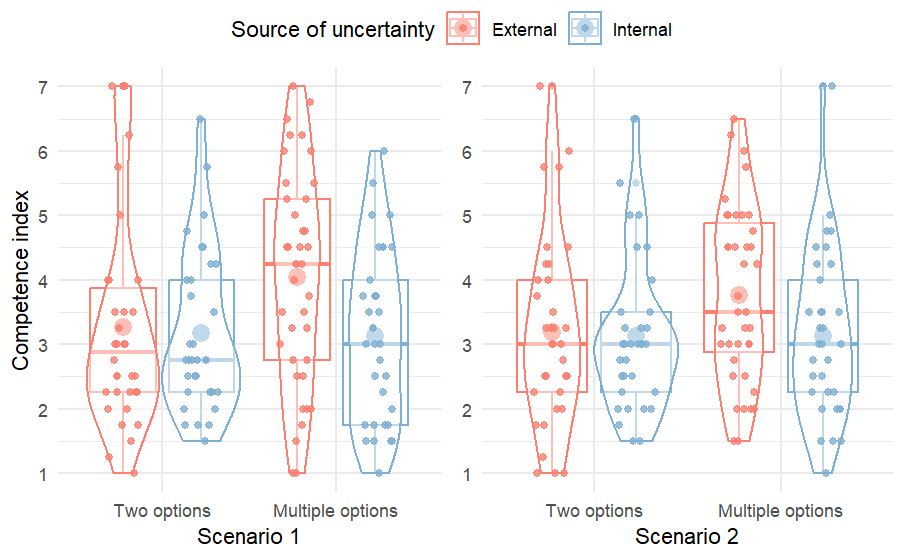


*Figure S2.* Ratings of leadership competence by condition and scenario, Supplemental Study S1. *Note*. Individual responses are shown as smaller dots. Violins display the distribution of responses. Boxplots display the median, first, and third quartiles. Larger dots show mean values.

Table S9. *Mean ratings of honesty, openness, and the extent to which a good reason for a choice was provided, in the second scenario of Supplemental Study S1. Standard deviations in parentheses.*

|  | Two options | | Multiple options | |
| --- | --- | --- | --- | --- |
|  | External  (n = 34) | Internal  (n = 33) | External  (n = 39) | Internal  (n = 33) |
| Honesty | 4.65 (1.94) | 5.00 (1.41) | 4.59 (1.85) | 5.61 (1.35) |
| Openness | 4.68 (2.01) | 5.09 (1.61) | 5.03 (1.72) | 5.97 (0.95) |
| Good reason provided | 1.79 (1.30) | 1.79 (1.24) | 2.10 (1.19) | 2.24 (1.68) |
| Honesty-index | 4.66 (1.80) | 5.05 (1.23) | 4.81 (1.56) | 5.79 (0.96) |


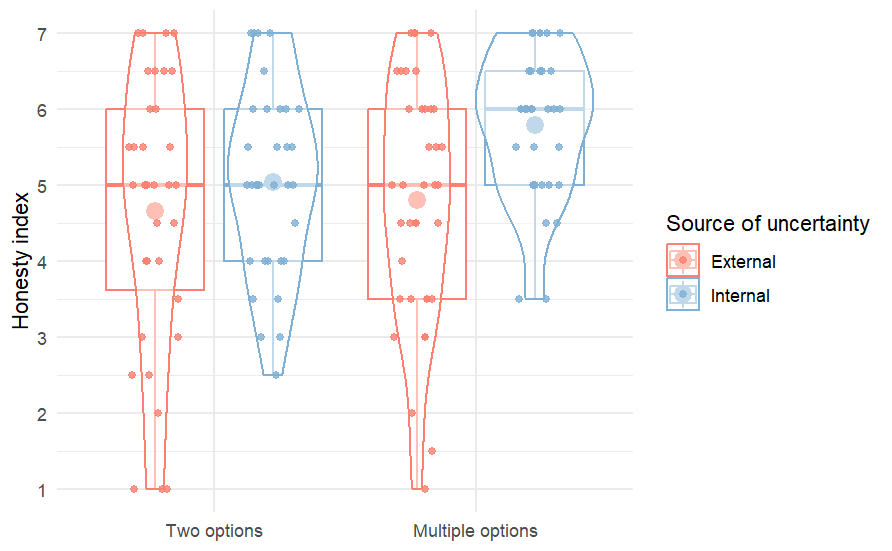


*Figure S3.* Ratings of honesty and openness (combined) by condition, Supplemental Study S1. *Note*. Individual responses are shown as smaller dots. Violins display the distribution of responses. Boxplots display the median, first, and third quartiles. Larger dots show mean values.

Ratings of both honesty and openness (only judged for the second scenario) showed an effect of the source of uncertainty (see Table S8). Since these ratings were (as expected) related to each other, *r* = .54, *p* < .001, we analyzed the combined score for these two variables^[[4]](#footnote-4)^. An ANOVA on this “honesty-index” showed an effect of source of uncertainty, *F*(1,135) = 7.83, *p* = .006, η_p_^2^ = .055, but no significant effect of number of options, *F*(1,135) = 3.32, *p* = .071, η_p_^2^ = .024, and no interaction, *F*(1,135) = 1.50, *p* = .223.^[[5]](#footnote-5)^ The effect of source of uncertainty was due to higher ratings for internal expressions (*M* = 5.42, *SD* = 1.16) than for external expressions (*M* = 4.74, *SD* = 1.67), *d* = 0.48 [0.14, 0.82], see also Figure S3.

Finally, for ratings of whether the leader provided a good reason for his choice, there were no statistically significant effects of either number of options, *F*(1,128) = 2.73, *p* = .101, η_p_^2^ = .020, nor of source of uncertainty or the interaction between the two factors, *F*’s < 1, *p*’s > .75. The overall score was very low (grand mean of 1.99 on a scale from 1 to 7), which seems a natural consequence of the fact that no particular reason for making the choice was provided in these scenarios.

**Discussion**

Supplemental Study S1 did not achieve the statistical power we aimed for, so the results must be interpreted with caution. Nevertheless, some interesting tendencies were observed. The main effect of source of uncertainty was not statistically significant in this study, even if competence ratings were somewhat higher for leaders using external rather than internal expressions. However, there was a statistically significant effect of source of uncertainty on perceived honesty, with leaders sharing their personal uncertainty rated as more honest and open than those pointing towards external uncertainty. This observed “honesty premium” for internal uncertainty indicates that different expressions of uncertainty may have different strengths and weaknesses. We did not find any statistically significant effects of number of options in the choice set.

# Considerations about pre-registration, error rates, sample size and power analysis

Four of the studies reported here (Study 2, 3, 4, and S1) were pre-registered, while Study 1 and 5 were not pre-registered. We note that results from Study 1 and 5 should thus be interpreted with more caution.

In all our studies, we opted for an alpha level of .05 and a statistical power of .80.^[[6]](#footnote-6)^ We chose these levels as they are general conventions in the field, even though there is a lot of debate about what the ideal levels for alpha and power should be (e.g., Lakens et al., 2018). This means that in our studies, the Type I error control is set at a stricter level than the Type II error control.

In conducting our studies, we faced resource constraints and had to take this into account with regards to sample sizes. We therefore followed Lakens’ (2022) suggestion and performed sensitivity analysis for all studies, except for Study 5, where it is more complicated to say what the relevant effect size of interest is. Thus, we report for each study which effect size we have an 80% chance to detect.

# Materials for all studies

## Study 1

**Innovation projects**

In a midsize tech company, there are two ongoing innovation projects, Project A and Project B. However, the projects are costly, and the company can only afford to invest in one of them. The projects are evaluated by an independent group of developers, and after the CEO of the company reads their report, he sends out an email to the two project groups, with the following conclusion:

*"The evaluation report shows that both projects have their pros and cons, and it is [I am] quite [un]certain which project has the greater promise. However, I have decided that we will invest in Project A."*

What is your impression of the CEO and his decision? Rate your agreement with the statements below.

|  | Disagree completely  (1) | (2) | (3) | (4) | (5) | (6) | Agree completely  (7) |
| --- | --- | --- | --- | --- | --- | --- | --- |
| The CEO seems competent |  |  |  |  |  |  |  |
| The CEO seems confident |  |  |  |  |  |  |  |
| The CEO put a lot of thought into the decision |  |  |  |  |  |  |  |
| The decision was difficult to make for the CEO |  |  |  |  |  |  |  |
| The CEO seems like a good leader |  |  |  |  |  |  |  |

[Page break]

As you remember, the email from the CEO concluded:

*"The evaluation report shows that both projects have their pros and cons, and it is [I am] quite [un]certain which project has the greater promise. However, I have decided that we will invest in Project A."*

One year after this decision, after large investments of work and money into Project A, it becomes clear that the project has been a success [failure], and that the investment has [not] paid off.
Please read the statements below and rate your agreement.

|  | Disagree completely  (1) | (2) | (3) | (4) | (5) | (6) | Agree completely  (7) |
| --- | --- | --- | --- | --- | --- | --- | --- |
| It was quite predictable what the outcome of the project would be |  |  |  |  |  |  |  |
| It was clear from the beginning that the project could go both ways |  |  |  |  |  |  |  |
| The CEO is responsible for the choice of the project |  |  |  |  |  |  |  |
| The CEO deserves credit [blame] for the outcome of the project |  |  |  |  |  |  |  |
| I would trust the CEO to make good decisions in the future |  |  |  |  |  |  |  |

[Page break]

**Bacteria outbreak**

There is an outbreak of multiresistant bacteria in two neighboring European countries. If the outbreak is not stopped, experts estimate that several hundred people could die within a year. The minister of health in one of the countries has to choose between two different strategies to handle the outbreak, Strategy A and Strategy B. After reading a research report about the two strategies, the minister makes the following statement:
*"Based on the most recent research, it is [I am] [un]certain which strategy will give the best result. I have decided that we should use Strategy A."*

 What is your impression of the minister of health and his decision? Rate your agreement with the statements below.

|  | Disagree completely  (1) | (2) | (3) | (4) | (5) | (6) | Agree completely  (7) |
| --- | --- | --- | --- | --- | --- | --- | --- |
| The minister seems competent |  |  |  |  |  |  |  |
| The minister seems confident |  |  |  |  |  |  |  |
| The minister put a lot of thought into the decision |  |  |  |  |  |  |  |
| The decision was difficult to make for the minister |  |  |  |  |  |  |  |
| The minister seems like a good leader |  |  |  |  |  |  |  |

[Page break]

As you remember, the minister made the following statement:

*"Based on the most recent research, it is [I am] [un]certain which strategy will give the best result. I have decided that we should use Strategy A."*

Six months after the decision to use Strategy A to handle the outbreak of multiresistant bacteria, it becomes clear that the strategy has largely been a success [failure]. The outbreak has [not] been contained, and in the neighboring country, which followed Strategy B, many more [fewer] patients have died.

Please read the statements below and rate your agreement.

|  | Disagree completely  (1) | (2) | (3) | (4) | (5) | (6) | Agree completely  (7) |
| --- | --- | --- | --- | --- | --- | --- | --- |
| It was quite predictable what the outcome of following Strategy A would be |  |  |  |  |  |  |  |
| It was clear from the beginning that following Strategy A could go both ways |  |  |  |  |  |  |  |
| The minister is responsible for the choice of the strategy |  |  |  |  |  |  |  |
| The minister deserves credit [blame] for the outcome |  |  |  |  |  |  |  |
| I would trust the minister to make good decisions in the future |  |  |  |  |  |  |  |

## Study 2

**Innovation projects**
  

In a midsize tech company, there are two ongoing innovation projects, Project A and Project B. However, the projects are costly, and the company can only afford to invest in one of them. The CEO of the company gathers information about the projects, and then sends out an email to the two project groups with the following conclusion:
  *"Both projects have their pros and cons, and it is [I am] quite [un]certain which project has the greater promise. However, I have decided that we will invest in Project A."*

 What is your impression of the CEO and his decision? Rate your agreement with the statements below.

|  | Disagree completely  (1) | (2) | (3) | (4) | (5) | (6) | Agree completely  (7) |
| --- | --- | --- | --- | --- | --- | --- | --- |
| The CEO seems competent |  |  |  |  |  |  |  |
| The CEO seems confident |  |  |  |  |  |  |  |
| The CEO put a lot of thought into the decision |  |  |  |  |  |  |  |
| The CEO seems like a good leader |  |  |  |  |  |  |  |

**Bacteria outbreak**
  
There is an outbreak of multiresistant bacteria in two neighboring European countries. If the outbreak is not stopped, experts estimate that several hundred people could die within a year. The minister of health in one of the countries has to choose between two different strategies to handle the outbreak, Strategy A and Strategy B. After gathering information about the two strategies, the minister makes the following statement:
  *"Both strategies have strengths and weaknesses, and it is [I am] quite [un]certain which strategy will give the best result. However, I have decided that we should use Strategy A."*

What is your impression of the minister of health and his decision? Rate your agreement with the statements below.

|  | Disagree completely  (1) | (2) | (3) | (4) | (5) | (6) | Agree completely  (7) |
| --- | --- | --- | --- | --- | --- | --- | --- |
| The minister seems competent |  |  |  |  |  |  |  |
| The minister seems confident |  |  |  |  |  |  |  |
| The minister put a lot of thought into the decision |  |  |  |  |  |  |  |
| The minister seems like a good leader |  |  |  |  |  |  |  |

## Study 3

**Sustainability strategy**
  

A construction company is looking to improve their sustainability, as a response to government regulations coming over the next decade, as well as requests from clients. The company is considering two different strategies. Strategy A involves the use of new experimental building materials, while Strategy B involves improving the recycling of materials from building sites.

The CEO of the construction company has hired a group of independent consultants to research the two strategies. After reading their report, the CEO makes the following announcement:
  *"It is clear that both strategies have their pros and cons, and based on the report, it is [I am] uncertain which strategy has the greater promise. So I have decided that more information should be gathered before I make my choice about which strategy we should use. [However, I have decided that we should use Strategy A.]"*

What is your impression of the CEO and his decision? Rate your agreement with the statements below.

|  | Disagree completely  (1) | (2) | (3) | (4) | (5) | (6) | Agree completely  (7) |
| --- | --- | --- | --- | --- | --- | --- | --- |
| The CEO seems competent |  |  |  |  |  |  |  |
| The CEO seems confident |  |  |  |  |  |  |  |
| The CEO put a lot of thought into the decision |  |  |  |  |  |  |  |
| The CEO seems like a good leader |  |  |  |  |  |  |  |

[Page break]

As you remember, the CEO stated that "it is [I am] uncertain which strategy has greater promise" and decided that it was necessary to gather more information about the two strategies. [As you remember, despite stating that "it is [I am] uncertain which strategy has greater promise", the CEO chose Strategy A. However, a few days later the CEO reconsidered and decided that it was necessary to gather more information about the two strategies.] He had the consultancy company do more research on the strategies, and after two months, they delivered a new report. With more information in place, what do you think is likely to happen?

Please read the statements below and indicate what you think will happen by selecting 1 for the most likely outcome, 2 for the next most likely outcome, and 3 for the least likely outcome.

______ It [The CEO] will become more uncertain which alternative has greater promise

______ It [The CEO] will become more certain which alternative has greater promise

______ The [CEO’s] uncertainty about which alternative has greater promise will not change

 [Page break]

The scenario you just read described a situation where there are two alternatives, but it [the CEO] is uncertain which alternative is better. Such situations are quite common in business settings. While it can sometimes be possible to gather more information or to postpone the decision, at other times a choice has to be made without further delay.
 
Now imagine that you are an employee in a company, and that the CEO has to make a choice between two alternatives, even though it [he] is uncertain which alternative is the best. In such a situation, which of the two possible courses of action described below would you prefer from the CEO?

- After making the choice, the CEO communicates that it [he] is quite uncertain which alternative is better
- After making the choice, the CEO downplays the [his] uncertainty and communicates that it [he] is quite certain which alternative is better

Please give a short explanation (1-3 sentences) why you would prefer this course of action from the CEO:

## Study 4

**Leaders making choices**

In a large tech company, there are two managers for different departments. These managers often make choices and have to communicate their choices to the employees. Below are two slightly different ways the two managers have announced recent choices about investing in ongoing projects. Please read the two statements carefully.

Manager A: "**It is very [somewhat] uncertain** which of the two projects has greater promise. However, I have decided that we will invest in Project X."
Manager B: "**I am very [somewhat] uncertain** which of the two projects has greater promise. However, I have decided that we will invest in Project Y."

Which of the two managers seems more competent?

- Definitely Manager A (1)
- (2)
- (3)
- (4)
- (5)
- (6)
- Definitely Manager B (7)

[Page break]

Here the same two statements are presented again. Take a look at them and then answer the question below.
 
Manager A: "**It is very [somewhat] uncertain** which of the two projects has greater promise. However, I have decided that we will invest in Project X."

Manager B: "**I am very [somewhat] uncertain** which of the two projects has greater promise. However, I have decided that we will invest in Project Y."

Which of the two managers seems more honest about a difficult decision?

- Definitely Manager A (1)
- (2)
- (3)
- (4)
- (5)
- (6)
- Definitely Manager B (7)

## Study 5

**Questions about work experience**

Please indiate your employment status.

- Working full-time
- Working part-time
- Unemployed
- Homemaker
- Student
- Retired

Which of the options below best describes your current position?

- Upper level management
- Mid-level management
- Lower level management (e.g., supervisor, team leader)
- Not currently in a management position, but was in a management position previously
- Have never held a management position

How much work experience do you have in total?

- 1-6 months
- 6-12 months
- Fill in the number of years below __________________________________________________

How much experience as a manager do you have?

- 1-6 months
- 6-12 months
- Fill in the number of years below __________________________________________________

Approximately how many people are employed in the organization which you work in?

- Less than 20
- 20-49
- 50-99
- 100-249
- 250-499
- 500-1000
- More than 1000

At work, how many people do you have authority to give instructions to?

- 1
- 2-3
- 4-6
- 7-10
- 10-20
- More than 20

Please write a short description of what your organization does (e.g., agriculture, advertisment, IT, finance, retail, energy, industry - whichever brief description you find the most informative).

**Making decisions as a manager**

You are invited to take part in this study because you have management experience. Managers often have to make different kinds of decisions, and in this study, we are interested in managers' decision making, and how they communicate with others (e.g., colleagues, stakeholders) about their decisions.

Please think about a time when you as a manager had to make a decision, but you were [it was] uncertain what was the best decision to make. In the box below, briefly describe the decision and why you were [it was] uncertain.

For the decision you just described, did you communicate to others (e.g., colleagues, stakeholders) that you were uncertain [there was uncertainty] about what would be the best decision? Choose the option below that best describes your behavior.

- I did not mention uncertainty - in fact I instead explicitly stated that I [it] was quite certain what was the best choice (1)
- I deliberately chose not to mention that I was uncertain [there was uncertainty] (2)
- I did not find it relevant to mention that I was uncertain [there was uncertainty] (3)
- I mentioned that I was uncertain [there was uncertainty], but downplayed the extent of uncertainty (4)
- I gave an honest portrayal of how uncertain I [it] was (5)
- I overstated the degree to which I was uncertain [there was uncertainty] (6)

When you were making the decision you described, what characterized the uncertainty associated with the decision? Please indicate your agreement with the statements below.

|  | Disagree completely  1 | 2 | 3 | 4 | 5 | 6 | Agree completely  7 |
| --- | --- | --- | --- | --- | --- | --- | --- |
| The uncertainty was due to external factors beyond my control |  |  |  |  |  |  |  |
| The uncertainty was related to my knowledge or beliefs about the decision |  |  |  |  |  |  |  |
| The uncertainty was an objective fact that would be apparent to other people |  |  |  |  |  |  |  |
| The uncertainty was a subjective feeling I had |  |  |  |  |  |  |  |

In this part of the survey, we are interested in your general beliefs when it comes to decision making as a manager.


There are undoubtedly many situations in which you as a manager have to make a decision, but you are [it is] uncertain which option is better. Sometimes it might be possible to gather more information or to postpone the decision, but at other times you have to make a choice without further delay. In such a situation, if you have to make a choice even though you are [it is] uncertain which option is better, which of the alternatives below would you generally prefer?

- When making my choice, I would be open about the fact that I am [it is] uncertain which option is better (1)
- When making my choice, I would not mention the fact that I am [it is] uncertain which option is better (2)
- When making my choice, I would explicitly state that I am [it is] quite certain which option is better (3)

In general, what are your beliefs about mentioning uncertainty to others in a work setting? Please read through the statements below and indicate your agreement with them.

If I reveal that I am uncertain [there is uncertainty] about a choice, I believe that people will see me as incompetent

- Disagree completely 1
- 2
- 3
- 4
- 5
- 6
- Agree completely 7

If I reveal that I am uncertain [there is uncertainty] about a choice, I believe that people will see me as honest and open

- Disagree completely 1
- 2
- 3
- 4
- 5
- 6
- Agree completely 7

If I reveal that I am uncertain [there is uncertainty] about a choice, people may think of me as indecisive

- Disagree completely 1
- 2
- 3
- 4
- 5
- 6
- Agree completely 7

If I reveal that I am uncertain [there is uncertainty] about a choice, I will not inspire confidence in my colleagues and others

- Disagree completely 1
- 2
- 3
- 4
- 5
- 6
- Agree completely 7

If I reveal that I am uncertain [there is uncertainty] about a choice, people will be more prepared for a variety of outcomes

- Disagree completely 1
- 2
- 3
- 4
- 5
- 6
- Agree completely 7

If I reveal that I am uncertain [there is uncertainty] about a choice, I will be held less accountable for a potential negative outcome

- Disagree completely 1
- 2
- 3
- 4
- 5
- 6
- Agree completely 7

In general, what would you prefer in the situations described below? Please read the descriptions and choose the option you would generally prefer.

When making a choice, I would prefer to say to others that

- I am uncertain which option is the best (1)
- it is uncertain which option is the best (2)

When making a choice, I would prefer to say to others that

- I am quite certain which option is the best (1)
- it is quite certain which option is the best (2)

## Supplemental Study S1

[The text below is translated to English from the original Norwegian]

**Innovation projects**  

A mid-sized IT-company currently has 2 [6] ongoing innvoation projects. However, the projects are expensive to maintain, and the company now has to decide which project it should invest the most in. An independent group of computer engineers is being hired to evaluate the projects and delivers a report. After the company’s CEO has read this report, he sends out an email to the innovation department with the following conclusion:

*"Both of the two [All of the six] projects have their pros and cons, and based on the report it is [I am] quite uncertain which project has the greatest potential. I have still decided that we should invest most of our funds in Project Z-45."*

How would you evaluate the CEO and the decision he made? Indicate how much you agree with the statements below on a scale from 1 (Disagree completely) to 7 (Agree completely).

|  | Disagree completely  (1) | (2) | (3) | (4) | (5) | (6) | Agree completely  (7) |
| --- | --- | --- | --- | --- | --- | --- | --- |
| The leader seems competent |  |  |  |  |  |  |  |
| The leader seems confident |  |  |  |  |  |  |  |
| The leader seems to have given a lot of thought to the decision |  |  |  |  |  |  |  |
| The leader seems like a good leader |  |  |  |  |  |  |  |

**Marketing of new products**  

A company producing foodstuffs has developed 2 [8] new products that is now going to be introduced to the market and is planning a marketing campaign. However, the budget is limited, and even though both [all] products will be advertised online, there are only funds to make a TV-commercial for one of the products. The leader of the marketing department has asked a project group to make a report about the potential of the products, and after reading the report, the leader sends out an email with the following conclusion:

*"Both of the two [All of the eight] products have potential, but based on the report it is[I am] quite uncertain which product will benefit the most from a TV campaign. I have still chosen that we should make a TV-commercial for Product A."*

How would you evaluate the leader of the marketing department and the decision he made? Indicate how much you agree with the statements below on a scale from 1 (Disagree completely) to 7 (Agree completely).

|  | Disagree completely  (1) | (2) | (3) | (4) | (5) | (6) | Agree completely  (7) |
| --- | --- | --- | --- | --- | --- | --- | --- |
| The leader seems competent |  |  |  |  |  |  |  |
| The leader seems confident |  |  |  |  |  |  |  |
| The leader seems to have given a lot of thought to the decision |  |  |  |  |  |  |  |
| The leader seems like a good leader |  |  |  |  |  |  |  |

[Page break]

As you remember, the leader of the marketing department sent out an email with the following conclusion:

*"Both of the two [All of the eight] products have potential, but based on the report it is[I am] quite uncertain which product will benefit the most from a TV campaign. I have still chosen that we should make a TV-commercial for Product A."*

Please evaluate the statements below on a scale from 1 (Disagree completely) to 7 (Agree completely).

|  | Disagree completely (1) | (2) | (3) | (4) | (5) | (6) | Agree completely (7) |
| --- | --- | --- | --- | --- | --- | --- | --- |
| The leader seems honest about difficult decisions |  |  |  |  |  |  |  |
| The leader does not seem afraid to admit uncertainty |  |  |  |  |  |  |  |
| The leader gave a good reason for his choice |  |  |  |  |  |  |  |

# References

Chernev, A., Böckenholt, U., & Goodman, J. (2015). Choice overload: A conceptual review and meta-analysis. *Journal of Consumer Psychology*, *25*(2), 333–358. https://doi.org/10.1016/j.jcps.2014.08.002

Faul, F., Erdfelder, E., Lang, A.-G., & Buchner, A. (2007). G*Power 3: A flexible statistical power analysis program for the social, behavioral, and biomedical sciences. *Behavior Research Methods*, *39*(2), 175–191. https://doi.org/10.3758/BF03193146

Lakens, D. (2022). Sample Size Justification. *Collabra: Psychology*, *8*(1), 33267. https://doi.org/10.1525/collabra.33267

Lakens, D., Adolfi, F. G., Albers, C. J., Anvari, F., Apps, M. A. J., Argamon, S. E., Baguley, T., Becker, R. B., Benning, S. D., Bradford, D. E., Buchanan, E. M., Caldwell, A. R., Van Calster, B., Carlsson, R., Chen, S.-C., Chung, B., Colling, L. J., Collins, G. S., Crook, Z., … Zwaan, R. A. (2018). Justify your alpha. *Nature Human Behaviour*, *2*(3), 168–171. https://doi.org/10.1038/s41562-018-0311-x

Tenney, E. R., Spellman, B. A., & MacCoun, R. J. (2008). The benefits of knowing what you know (and what you don’t): How calibration affects credibility. *Journal of Experimental Social Psychology*, *44*(5), 1368–1375. https://doi.org/10.1016/j.jesp.2008.04.006

1. Several participants only responded to one or two out of the three ratings for this question. To make results comparable across the three options, we only analyzed responses from those who had filled in all three answers. However, the same pattern of results occurred when all responses were included.. [↑](#footnote-ref-1)
2. We pre-registered that we would aim for at least 180 participants, but failed to achieve this goal. [↑](#footnote-ref-2)
3. Analyzing results with separate index variables for each scenario in a 2 x 2 x 2 ANOVA with scenario as within-subjects factor gave identical results, with no statistically significant effects of scenario, all p’s > .32. [↑](#footnote-ref-3)
4. Analyzing the results separately gave similar results, but for the openness variable there was also an effect of the number of options, *F*(1,135) = 4.92, *p* = .028, η_p_^2^ = .035 with higher ratings when there were many options (*M* = 5.46, SD = 1.49) than when there were two (*M* = 4.88, *SD* = 1.82). [↑](#footnote-ref-4)
5. Levene’s test indicated unequal error variances across groups, *F*(3,135) = 4.55, *p* = .005. Analysis with non-parametric alternatives gave similar results so we chose to report the ANOVA. [↑](#footnote-ref-5)
6. Note that in the pre-registrations we incorrectly describe that we aim for a beta of .80. This should of course have been power (1 – beta). [↑](#footnote-ref-6)
